# Design and development of an oral remdesivir derivative VV116 against SARS-CoV-2

**DOI:** 10.1038/s41422-021-00570-1

**Published:** 2021-09-28

**Authors:** Yuanchao Xie, Wanchao Yin, Yumin Zhang, Weijuan Shang, Zhen Wang, Xiaodong Luan, Guanghui Tian, Haji A. Aisa, Yechun Xu, Gengfu Xiao, Jia Li, Hualiang Jiang, Shuyang Zhang, Leike Zhang, H. Eric Xu, Jingshan Shen

**Affiliations:** 1 Shanghai Institute of Materia Medica, Chinese Academy of Sciences, Shanghai, China; 2 State Key Laboratory of Virology, Wuhan Institute of Virology, Center for Biosafety Mega-Science, Chinese Academy of Sciences, Wuhan, Hubei China; 3 School of Medicine, Tsinghua University, Beijing, China; 4 Department of Cardiology, Peking Union Medical College Hospital, Peking Union Medical College and Chinese Academy of Medical Sciences, Beijing, China; 5 Tsinghua-Peking Center for Life Sciences, Tsinghua University, Beijing, China; 6 Vigonvita Life Science Co., Ltd., Suzhou, Jiangsu China; 7 University of Chinese Academy of Sciences, Beijing, China; 8 Key Laboratory of Plant Resources and Chemistry in Arid Regions, Xinjiang Technical Institute of Physics and Chemistry, Chinese Academy of Sciences, Urumqi, Xinjiang China; 9 State Key Laboratory of Drug Research, Shanghai Institute of Materia Medica, Chinese Academy of Sciences, Shanghai, China

**Keywords:** Cell biology, Molecular biology, Immunology

Dear Editor,

Since the declaration of COVID-19 as a global pandemic on March 11, 2020, this pandemic has been circulating for 17 months throughout the world, leading to more than 200 million infections and nearly 4.4 million deaths as of August 15, 2021. The pathogen of COVID-19 is a novel coronavirus named SARS-CoV-2, which shares ~79% genome sequence identity with SARS-CoV.^1^ At the early stage of the COVID-19 outbreak, SARS-CoV-2 caused great panic in the hardest-hit areas due to its high transmissibility and pathogenicity. To fight the COVID-19 crisis, drug repurposing was immediately pursued in order to find potential therapeutics. Until now, though some treatment options are available, there still lack effective antiviral agents. Vaccination is considered as the best way to control this global pandemic, but the emerging SARS-CoV-2 variants pose a great challenge to the protective efficacy of the existing vaccines. Hence, in the long run, finding an effective antiviral medication against the SARS-CoV-2 infection is a pressing need.

Great efforts have been put into the development of novel drugs against SARS-CoV-2 infection. Currently, remdesivir (RDV) (Fig. 1a), an intravenously administered nucleotide prodrug, is the only approved anti-SARS-CoV-2 drug. However, it displays limited therapeutic efficacy especially for severe COVID-19 cases.^2^ Nucleoside analogs are an important class of antivirals acting by interfering with the viral polymerases that are highly conserved in the active center. Besides RDV, two oral nucleoside analogs, molnupiravir (EIDD-2801)^3^ and AT-527,^4^ have entered phase II/III clinical studies. Herein, we reported the discovery of an oral anti-SARS-CoV-2 nucleoside candidate, VV116, which displays favorable drug-like properties, and is going to be evaluated in clinical study for treating COVID-19.

**Fig. 1:**
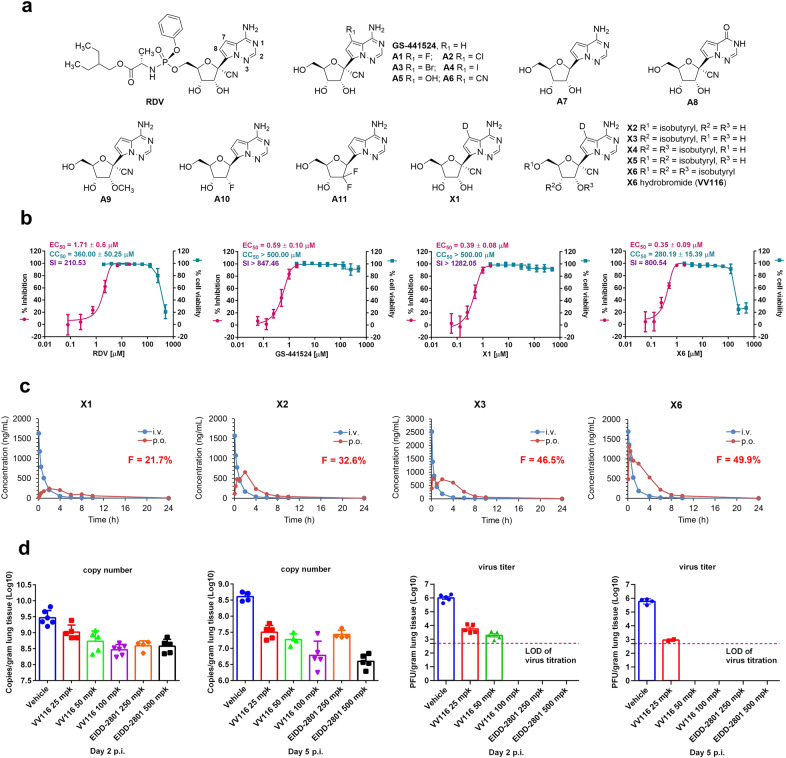
Discovery of VV116 as a promising orally administered anti-SARS-CoV-2 nucleoside drug candidate. **a** The chemical structures of RDV, GS-441524, GS-441524 derivatives A1–A11, the 7-deuterated GS-441524 analog X1, and the isobutyrate ester prodrugs X2–X6. **b** Inhibition of SARS-CoV-2 replication and cellular toxicity by RDV, GS-441524, X1 and X6 in Vero E6 cells. **c** Mean plasma concentrations of X1 following single intravenous (2.0 mg/kg X1 equivalent dose) and oral (10.0 mg/kg X1 equivalent dose) administration of X1, X2, X3 or X6 to SD rats (*n* = 3 per group). **d** Viral RNA levels and infectious virus titers in lung tissues of control (vehicle-treated), VV116- and EIDD-2801-treated mice on day 2 and day 5 p.i. For VV116, there were three dosage groups (25 mg/kg, 50 mg/kg and 100 mg/kg, mg/kg = mpk), and for EIDD-2801, there were two dosage groups (250 mg/kg and 500 mg/kg). Error bars indicate SEM.

At the onset of the COVID-19 outbreak, we conducted a fast in vitro screening for anti-SARS-CoV-2 activity of various nucleoside/nucleotide analogs (listed in Supplementary information, Table S1) in Vero E6 cells. Most of these compounds are known antiviral agents that show inhibitory activities against one or several viruses. Among them, we discovered that only RDV and its parent nucleoside (GS-441524) could remarkably inhibit the replication of SARS-CoV-2 at 5.0 µM. Determination of their EC_50_ values indicated that GS-441524 with an EC_50_ of 0.59 µM was a stronger viral replication inhibitor than RDV in Vero E6 cells (Fig. 1b). RDV is a phosphoramidate prodrug of GS-441524, originally designed for enhancing the antiviral activity against hepatitis C virus.^5^ This kind of prodrug exhibits liver-targeting property, and may not be suitable for application in nucleoside-based antiviral therapy for COVID-19 since lungs are the most affected organs. Therefore, we chose GS-441524 as the parent structure for modifications to achieve improved druggability against SARS-CoV-2.^6^

A previous study showed that substituents at the 7-position (purine numbering) of the pyrrolotriazine base had a great influence on the antiviral activities of the pyrrolotriazine C-nucleosides.^7^ Hence, we modified GS-441524 by introducing different groups (halogen, hydroxyl or cyano) at this site. Among the six synthesized derivatives (A1–A6, Fig. 1a), only the fluoro-substituted nucleoside A1 showed moderate anti-SARS-CoV-2 activity with an EC_50_ of 3.3 µM (Supplementary information, Table S2), which was ~5-fold less potent than GS-441524. Structural modifications at the 1′- and 2′-positions of this nucleoside were also conducted. We found that removal of the 1′-CN of GS-441524 afforded a highly cytotoxic nucleoside A7, and conversion of the 6-amino group to hydroxyl group or methylation of the 2′-α-OH abolished the antiviral activity (A8 and A9). Two other derivatives (A10 and A11) derived from GS-441524 by removing the 1′-CN and replacing the 2′-α-OH with a α-fluoro or a 2′,2′-difluoro group did not exhibit anti-SARS-CoV-2 activity, either. From the above result, it seemed that the frequently-used structural modifications were not tolerated on this nucleoside, and subtle changes would lead to a significant decrease or loss of antiviral activity.

GS-441524 has an adenine-mimicking pyrrolotriazine base that is characterized by an electron-rich double bond and a 1,2,4-triazine ring. This unnatural base might be more vulnerable to enzymatic degradation through oxidation of the double bond or ring opening of the triazine moiety. Therefore, we attempted to modify GS-441524 by deuteration that may confer potential pharmacokinetic (PK) benefits. Due to the synthetic difficulties, we only obtained the 7-deuterated derivative X1 (Fig. 1a). This nucleoside displayed strong antiviral activity with an EC_50_ value of 0.39 ± 0.08 µM (Fig. 1b), and was found to have poor water solubility and liposolubility, which likely led to its low oral bioavailability in rats (*F* = 21.7%; Fig. 1c and Supplementary information, Table S3). To improve the oral bioavailability, several ester prodrugs of X1 were designed by introducing mono-, di- and tri-esters at the 2′-, 3′-, and 5′-positions of the ribose fragment. PK studies in rats showed that the 3′-isobutyrate prodrug X3 had a moderate bioavailability (*F* = 46.5%; Fig. 1c and Supplementary information, Table S3), which was about 2.1 times of that of X1, and 1.4 times of that of the 5′-isobutyrate prodrug X2 (*F* = 32.6%; Fig. 1c and Supplementary information, Table S3). However, the subsequent PK study in monkeys did not reveal a satisfying bioavailability (*F* = 18.4%; Supplementary information, Table S4) for X3. Therefore, this prodrug was not further evaluated. The 2′,3′-di-isobutyrate ester X4 (Fig. 1a) was obtained as a gummy substance, and then subjected to salt formation. The resulting two solids, the hydrochloride and the sulfate salts of X4, were highly hydroscopic, which was not favorable for pharmaceutical application. For the 3′,5′-di-isobutyrate ester X5 (Fig. 1a), it suffered from an inevitable impurity due to the 3′-isobutyryl group migration to the 2′-position. The tri-isobutyrate ester X6 (Fig. 1a) had a good oral bioavailability (*F* = ~50%) in rats (Fig. 1c and Supplementary information, Table S3), and was superior to X3 in terms of the plasma concentration and the plasma exposure of the parent nucleoside X1. Because X6 was difficult to be crystallized, an intensive salt screening of X6 was performed. Among all the solids obtained, the hydrobromide salt (VV116) was finally identified as the most qualified candidate. VV116 was not hygroscopic, and showed a good chemical stability over high temperature (60 °C), lights, or high humidity for weeks. This salt had a remarkably improved oral bioavailability (*F* = ~80%; Supplementary information, Table S6) in rats in the subsequent preclinical study.

Then, we evaluated the anti-SARS-CoV-2 efficacy of orally administered VV116 (25 mg/kg, 50 mg/kg or 100 mg/kg, BID) in hACE2-transduced mice^8^ with EIDD-2801 as the positive control (250 mg/kg or 500 mg/kg, BID) (Supplementary information, Fig. S4). VV116 presented a dose-dependent efficacy in reducing the viral RNA copies and infectious virus titers in the lungs (Fig. 1d). At day 2 post infection (p.i.) when viral loads peaked, there was a 0.5 log_10_, 0.70 log_10_, and 1.0 log_10_ reduction in the RNA copies for the three VV116 groups, respectively, and at day 5 p.i., more prominent efficacy was observed (1.1 log_10_, 1.3 log_10_ and 1.7 log_10_ decrease in the RNA copies, respectively). With respect to the infectious virus titers, treatment of VV116 at a low dose (25 mg/kg) resulted in a 2.0 log_10_ reduction at day 2 p.i., and a 3.0 log_10_ reduction at day 5 p.i. The medium dose (50 mg/kg) of VV116 exhibited a stronger activity, and decreased the virus titers below the detection limit at day 5 p.i. For the high dose (100 mg/kg) of VV116 and the two doses of EIDD-2801 (250 mg/kg or 500 mg/kg), the virus titers were reduced below the detection limit at both day 2 and day 5 p.i. Histopathology examination of lung tissues revealed that there was moderate interstitial pneumonia in vehicle-treated mice at day 2 p.i. characterized with thickened alveolar septa, infiltration of inflammatory cells and necrotic debris, while the area and degree of interstitial inflammatory lesions in 100 mg/kg VV116-treated mice were significantly improved (Supplementary information, Fig. S5 and Table S5).

Scalable synthesis of X1 was successfully achieved using deuterium gas as the deuterating agent, thus ensuring the supply of VV116 required for preclinical evaluation. VV116 functioned by targeting the viral RNA-dependent RNA polymerase through its nucleoside triphosphate form with an IC_50_ of 0.67 ± 0.24 μM (Supplementary information, Fig. S2). The preclinical safety evaluation showed that the maximal tolerated single doses of VV116 were at least 2.0 g/kg and 1.0 g/kg in rats and Beagle dogs, respectively. For the 14 days repeated dose toxicity studies, the no observed adverse effect levels (NOAELs) were 200 mg/kg and 30 mg/kg in rats and dogs, respectively (details shown in Supplementary information). VV116 and X1 exerted little effect on hERG current, and showed no mutagenicity according to the results of the Ames test and micronucleus test of bone marrow cells. PK results showed that VV116 had high oral bioavailability, reaching 80% and 90% in rats and dogs, respectively (Supplementary information,  Tables S6–S11). The key metabolite X1 was widely distributed in rat tissues (Supplementary information, Fig. S3) of the intestine, lung, kidney, liver, heart, and brain, most of which are the preferred targets of SARS-CoV-2. This property of VV116 would confer potential advantages for the treatment of SARS-CoV-2 infection.

The above preclinical results prove VV116 as a safe and effective oral nucleoside drug candidate against SARS-CoV-2. The clinical studies of VV116 will be fast-tracked considering the tremendous threat caused by the rapid spread of SARS-CoV-2 variants.

## Supplementary information

Supplementary information
