# Supplementary material for: Design and development of an oral remdesivir derivative VV116 against SARS-CoV-2
Source: Cell Res. 2021 Sep 28;31(11):1212–4. doi: 10.1038/s41422-021-00570-1 (PMC8477624; doi:10.1038/s41422-021-00570-1)
Supplement: Supplementary file 1 — Supplementary information [file 41422_2021_570_MOESM1_ESM.pdf]

Supplementary Information, Materials and Methods

Cell lines and viruses

African green monkey kidney Vero E6 cells (ATCC-1586) were maintained in Dulbecco's modified Eagle's medium (DMEM) with 10% fetal bovine serum (FBS) and 1% penicillin–streptomycin antibiotics. Cells were kept at 37 °C in a 5% CO_2 atmosphere. The strains 2019-nCoV-WIV04 and B.1.351 of SARS-CoV-2 were obtained from National Virus Resource Center, and were propagated in Vero E6 cells. OC43-CoV was obtained from Wuhan University, and was propagated in RD cells. All experiments with authentic SARS-CoV-2 viruses were carried out in the Biosafety Level 3 facility of the Wuhan Institute of Virology, Chinese Academy of Sciences (CAS).

Antiviral activity and cytotoxicity assays

In our study, Vero E6 cells were pre-seeded to 48-well plates (50,000 cells/well) for 16-18 h, and treated with medium containing gradient concentration of nucleoside analogs at 100 μL/well for 1 h. Then, the cells were inoculated with SARS-CoV-2 at multiplicity of infection (MOI) of 0.01. One hour later, the supernatant was removed, and cells were washed with PBS, and treated with fresh medium containing gradient concentration of nucleoside analogs at 200 μL/well. At 24 h post infection, the cell supernatant was collected, antiviral activities were evaluated by quantification of viral copy numbers in the cell supernatant via real-time fluorescence quantitative PCR (qRT-PCR) as described in our previous study[1]. The inhibition rate of nucleoside analogs was calculated based on the viral copy number, and the 50% effective concentration (EC_{50}) was calculated with Graphpad Prism software 8.0. At least three independent experiments were performed.

The antiviral activity of nucleoside analogs against OC43-CoV was evaluated via immunofluorescence assay. In this assay, RD cells were pre-seeded to 96-well plates (30,000 cells/well) for 24 h followed by the treatment with medium containing gradient concentration of nucleoside analogs at 100 μL/well for 1 h. Then, OC43-CoV was directly added into the medium at an MOI of 0.1 until the end of the experiment. At 24 h post infection, the supernatant was discarded, and the infected cells were fixed and then probed with rabbit sera against the NP of OC43-CoV as the primary antibody. Afterwards, cells were incubated with Alexa 488-labeled goat anti-rabbit IgG (1:500, Abcam) and the nuclei were stained with DAPI. Finally, the 96-well plates with immunofluorescence were imaged on an Operetta high-content imaging system (PerkinElmer), and the percentages of infected and DAPI-positive cells were

calculated using the associated Harmony 3.5 software.

For cytotoxicity measurement, Vero E6 cells were added to a 96-well plate (20,000 cells/well), and added with medium containing gradient concentration of nucleoside analogs at 100 μL/well next day. The cytotoxicity was determined after 24 h using the CCK8 assay kit, and CC_{50} values of nucleoside analogs were calculated with Graphpad Prism software 8.0.

Fluorescence-based assay for SARS-CoV-2 RdRp activity

The detection of RNA synthesis by SARS-CoV-2 RdRp was established with the fluorescent dye QuantiFluor® dsDNA System (Promega), which bound dsRNA but not ssRNA template molecules. The fluorescence emitted was recorded using a BioTek Synergy H1 with excitation and emission filters at 504 and 531 nm, respectively. The assay recorded the synthesis of dsRNA in a reaction using a self-primer RNA as a template and ATP as the natural substrate, and the self-primer RNA was synthesized by Genscript Biotech Corporation with the sequences of 5'-bio-UUUUUUUUUUUUUUUUUUUUUUUUUUUUUUUAACAGGUUCUAGAACCUGUU-3'. Reactions were performed in individual wells of black 96-well low volume round bottom plates with the total reaction volume of 20 μl. The standard reaction contained 20 mM Tris-HCl, pH 8.0, 10 mM KCl, 6mM $MgCl_2$, 20 μM ATP, 0.2 μM self-primer RNA and 0.01% Trition-X100 with 15 μg/ml SARS-CoV-2 RdRp. After incubation of the SARS-CoV-2 RdRp with RNA and different concentrations of the nucleoside triphosphate of VV116 (116-NTP) for 10 min at room temperature, ATP was added and incubated for another 30 min. Then, the 20 μl reaction system was added to 200 μl RNA detection system and incubated for 10 min at room temperature, and the fluorescence was recorded immediately. The reaction with increasing concentrations of 116-NTP was set as Experience group; the reaction without 116-NTP was set as Control group; the reaction with only RNA and ATP was set as Blank A group; while the reaction with only SARS-CoV-2 RdRp was set as Blank B group. Fluorometric results were expressed as mean ± SD. The inhibition rate at each concentration point was calculated by the formula of (Control group- Experience group)/ (Control group- Blank A group- Blank B group), and the IC_{50} values were obtained by fitting the inhibition rate to the concentration-response equation with GraphPad Prism.

In vivo antiviral efficacy of VV116 in mice

The BALB/c mice were bred and maintained in specific pathogen free (SPF) environment at the Laboratory Animal Center of Wuhan Institute of Virology, CAS. Female mice at six to eight weeks-old were used in our study. The animal experiments

were conformed to the use and care of laboratory animals and approved by the Institutional Review Board of the Wuhan Institute of Virology, CAS.

Animals were divided into six groups (n = 10 for each group), the vehicle group, the group receiving VV116 25 mg/kg orally bis in die (b.i.d.) (VV116 25 mpk for short), the group receiving VV116 50 mg/kg orally b.i.d. (VV116 50 mpk for short), the group receiving VV116 100 mg/kg orally b.i.d. (VV116 100 mpk for short), the group receiving EIDD-2801 250 mg/kg orally b.i.d. (EIDD-2801 250 mpk for short), and the group receiving EIDD-2801 500 mg/kg orally b.i.d. (EIDD-2801 500 mpk for short). Mice were anesthetized by intraperitoneal injection of 2.5% Avertin (20 μL/g body weight) and transduced intranasally with 50 μL 5×10^9 PFU/ml of Ad5-ACE2. Five days later (day 0), mice were anesthetized and infected with 50 μL 2×10^6 PFU/ml of SARS-CoV-2. One hour after SARS-CoV-2 infection, mice were treated with vehicle (40%PEG400+10% Kolliphor® HS15+50% ultrapure water), VV116 or EIDD-2801 according to group description as described above. Mice were observed for clinical signs daily from day 0 to day 5. At day 2, half of mice were sacrificed. The lung tissues from euthanized mice were homogenized with DMEM, the homogenized tissues were centrifuged at 3000 rpm for 10 min at 4 °C, and RNA was extracted from the supernatant by RNeasy Mini Kit (Qiagen, 74104) for real-time quantitative PCR (RT-PCR) testing. The virus titers were determined by plaque assay following a general procedure. At day 5, the remaining sacrificed or dead mice were dissected and lungs were treated as described above for determining the RNA copies and virus titers.

For histopathologic analysis, another experiment with a 2-day treatment regime was conducted. 10 animals were divided into three groups, the vehicle group (n = 4), the group receiving VV116 50 mg/kg orally b.i.d. (n = 3) and the group receiving VV116 100 mg/kg orally b.i.d. (n = 3). The experiment was performed following the same procedure as described above. At day 2, all mice were sacrificed to collect the lungs. The Left Lung tissues were fixed with 4% paraformaldehyde for histopathologic examination. The right lungs were homogenized, and the numbers of viral RNA copies were determined by RT-PCR.

Pharmacokinetic studies (for screening)

The PK studies in SD rats were conducted at Suzhou HQ Bioscience Co., Ltd.. SD rats (N = 3 for each group) were fasted for 12 h before dosing. Each compound dissolved in DMSO-enthanol-PEG300-saline (5/5/40/50, v/v/v/v) was administered orally at 34.2 μmol/kg (10 mg/kg for X1, 12.4 mg/kg for X2, 12.4 mg/kg for X3, and 17.2 mg/kg for X6) and intravenously at 6.8 μmol/kg (2 mg/kg for X1, 2.5 mg/kg for X2, 2.5 mg/kg for X3, and 3.4 mg/kg for X6), respectively. Blood samples were collected from the jugular vein into

EDTA-K2 tubes at various time points post-dose. Serum samples were obtained following general procedures and the concentrations of analytes in the supernatant were analyzed by LC-MS/MS.

The pharmacokinetics of **X2** and **X3** in cynomolgus monkey were investigated at Shanghai Medicilon Inc. following single intravenous intraperitoneal injection (5 mg/Kg) and oral gavage (10 mg/Kg). Prior to oral administration, the animals were fasted overnight (10-18 hours), and food supply to the animals dosed orally was resumed 4 hours post-dose. The blood was taken via femoral vein, 1.0 mL/time point into tubes containing K2-EDTA and stored on ice until centrifuged. Serum samples were obtained following general procedures and the concentrations of analytes in the supernatant were analyzed by LC-MS/MS.

Preclinical pharmacokinetic studies

The preclinical PK studies of VV116 in two species (SD rats and Beagle dogs) were conducted at Suzhou HQ Bioscience Co., Ltd. according to the NMPA "Guidelines for Non-clinical Pharmacokinetics studies", May 2014.

The PK study in SD rats: 30 animals divided into five groups (N = 6 for each group with 3 male and 3 female) were fasted for 12 h before dosing. Animals in Group 1 were administered intravenously with VV116 (dissolved in DMSO/EtOH/PEG300/0.9%NaCl, 5/5/40/50, v/v/v/v) at a dose of 10 mg/kg (calculated as the free base form). For Group 2-4, VV116 (dissolved in 40%PEG400+10% Kolliphor® HS15+50% ultrapure water) was administered orally at 10 mg/kg, 30 mg/kg and 90 mg/kg, respectively, and the animals in Group 5 received multiple oral doses of VV116 at 30 mg/kg/d for seven days. Blood samples were collected from the jugular vein into EDTA-K2 tubes at various time points post-dose. Serum samples were obtained following general procedures and the concentrations of analytes in the supernatant were analyzed by LC-MS/MS.

The PK study in Beagle dogs: 18 animals divided into three groups (N = 6 for each group with 3 male and 3 female) were fasted for 12 h before dosing. For the first two groups, VV116 (dissolved in 40%PEG400+10% Kolliphor® HS15+50% ultrapure water) was administered orally at 10 mg/kg and 20 mg/kg (calculated as the free base form), respectively, and animals in the third group received multiple oral doses of VV116 at 20 mg/kg/d for seven days. After 1 week of washout period, VV116 was given to the first two group dogs by intravenous route at 10 mg/kg dissolved in DMSO/EtOH/PEG300/0.9% NaCl (5/5/40/50, v/v/v/v) and orally at 40 mg/kg, respectively. Blood samples were collected from the forelimb vein into EDTA anticoagulant tubes at various time points post-dose. Following general procedures, the serum samples were obtained and the concentrations of analytes in the supernatant were analyzed by LC-MS/MS.

Tissue distribution

Thirty SD rats were randomly divided into five groups (n = 6, half male and half male). All rats were intragastrically administered with a single dose of 30 mg/kg VV116. At 0, 0.25, 2, 6, 24 h post-dosing, the rats were anesthetized, and tissues including brain, heart, liver, spleen, lung, kidney, pancreas, testis, ovaries, uterus, stomach, small intestine, adrenal gland, skin, thymus, skeletal muscle, and abdominal adipose tissue were harvested. Blood samples were collected, and part of them were centrifuged to obtain the plasma. Tissue samples were individually homogenized, and the concentrations of **X1** in plasma, whole blood and tissue homogenates were analyzed by LC-MS/MS.

Metabolism

Metabolites of VV116 in SD rats (plasma, urine, feces and bile) and in Beagle dogs (plasma) were analyzed by UPLC-UV/Q-TOFMS after a single intragastric administration of VV116. The bile, urine and feces samples of rats were obtained in the excretion study; the rat plasma sample was obtained in the tissue distribution study, and the plasma sample of Beagle dogs was obtained in the PK study.

Preclinical safety evaluation of VV116

The single dose oral toxicity study and the 14-day repeated dose oral toxicity study in two species (SD rats and beagle dogs) were conducted at Center of Drug Safety Evaluation and Research, SIMM, CAS adhering to 2017 NMPA "Good Laboratory Practice for Non-clinical Laboratory Studies". The hERG safety assay, Ames test, rat micronucleus assay, and chromosome aberration test were conducted according to NMPA and ICHS7B guidelines.

The single dose oral toxicity study in rats: Groups of male and female SD rats (5 animals/sex/group) received a single oral gavage of VV116 at dose of 0 (40%PEG400+10% HS15+50% ultrapure water; vehicle control), 200 (low), 600 (mild) and 2000 mg/kg (high), and were observed for 14 days post-dose before sacrificed on Day 15. All animals were alive before sacrificed and no abnormal clinical signs were observed during the experimental period at all the doses. There were no drug-related significant changes in body weight and food consumption compared with the vehicle group. After dissection, no morphological change was observed. The acute maximum tolerated single oral gavage dose in SD rats was at least 2000 mg/Kg.

The single dose oral toxicity study in dogs: Groups of male and female Beagle dogs (1 animals/sex/group) received a single oral gavage of VV116 at dose of 0 (vehicle control), 50 (low), 250 (mild) and 1000 mg/kg (high), and were observed for 14 days post-dose before sacrificed on Day 15. In this study, no drug related findings were noted at the low or mild dose. At the high dose, vomiting, soft stool and body weight loss were observed in one male dog on Day 2. There was no drug-related clinical morphological change in all the

groups. The acute maximum tolerated single oral gavage dose in Beagle dogs was at least 1000 mg/Kg.

The 14-day repeated dose oral toxicity study in rats: Groups of male and female SD rats (15 animals/sex/group) received repeated oral doses of VV116 at 0 (vehicle control), 100 (low), 200 (mild) and 500 mg/Kg/d (high) for 14 days. Two-thirds of animals (10 animals/sex/group) were sacrificed on Day 15 and after a recovery period of 14 days, the other animals were sacrificed on Day 29. In this study, no obvious drug related changes were seen at the low and mild doses. At the high dose, adverse effects were observed including changes in hematological and serum chemistry parameters, increased urinary crystals, trivial or mild diffuse hypertrophy of bilateral thyroid follicular epithelium, trivial or mild hyperplasia of tracheal mucosa epithelium, and chronic active inflammation of tracheal mucosa. Most of the changes were reduced or reversed after a 14-day recovery period. Taken together, dose level of 200 mg/Kg may be considered a NOAEL.

The 14-day repeated dose oral toxicity study in dogs: Groups of male and female Beagle dogs (5 animals/sex/group) received repeated oral doses of VV116 at 0 (vehicle control), 30 (low), 100 (mild) and 250 mg/Kg/d (high) for 14 days. Three-fifths of animals (3 animals/sex/group) were sacrificed on Day 15 and after a recovery period of 14 days, the other animals were sacrificed on Day 29. All animals were alive before sacrificed, and no drug related changes were seen at the low dose. At the high dose, pathological changes were observed in brains, eyes, pancreas, livers, thymuses, epididymides, gullets and stomachs. At the mild dose, increased ALT and decreased TG were noted. Most of the changes were reversed after a 14-day recovery period for the mild and high dose group. In this study, the NOAEL was 30 mg/Kg.

Synthesis of A1-A11

A7 and **A10** are known compounds, which could be synthesized according to the reported methods[2,3], and the synthesis of other compounds was reported elsewhere[4].

Synthesis of X1-X6

The materials and reagents were commercially available and solvents, if necessary, were purified and dried by standard methods. ^{1}H-NMR and ^{13}C-NMR spectra were determined on a Brucker 400 Hz, Brucker 500 Hz or Brucker 600 Hz instrument. ESI-MS was determined on Finnigan^{TM} LTQ^{TM} (*Thermo Fisher Scientific*, Bremen, Germany) linear ion trap mass spectrometer. All reactions were monitored by thin-layer chromatography (TLC) on 25.4×76.2 mm silica gel plates (GF-254). The final compounds possessed a HPLC purity of ≥ 95%.

Preparation of X1

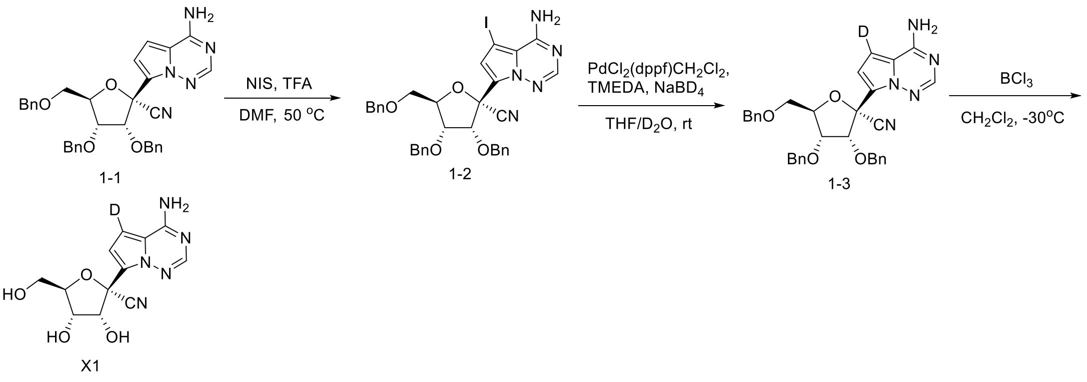

Compound **1-1** was prepared according to the previously reported method[5]. To a solution of **1-1** (20 g, 35.6 mmol) in DMF (70 mL), NIS (8.8 g, 39.2 mmol) and CF_3COOH (0.81 g, 7.1 mmol) were added. The mixture was stirred at 50 °C under N_2 protection for 1 h, then poured into a solution of Na_2SO_3 (9.0 g, 71.2 mmol) and Na_2CO_3 (15.1 g, 142.4 mmol) in water (150 mL), and extracted with ethyl acetate. The exact was washed with brine, dried over Na_2SO_4, and concentrated under vacuum. The resulting crude product was slurried in isopropyl ether and filtered to give **1-2** as a white solid (21.5 g, yield 88%). ^{1}H NMR (400 MHz, $CDCl_3$) δ 7.86 (s, 1H), 7.42 – 7.16 (m, 15H), 7.01 (s, 1H), 6.22 (s, 2H), 4.94 – 4.85 (m, 2H), 4.71 (d, *J* = 5.0 Hz, 1H), 4.62 – 4.52 (m, 2H), 4.51 – 4.41 (m, 2H), 4.32 (d, *J* = 12.0 Hz, 1H), 4.03 (dd, *J* = 7.0, 4.9 Hz, 1H), 3.82 (dd, *J* = 11.0, 3.3 Hz, 1H), 3.62 (dd, *J* = 11.0, 3.7 Hz, 1H).

Compound **1-2** (1.77 g, 2.57 mmol) was added to anhydrous tetrahydrofuran (20 mL) and deuteroxide (2 mL), and then the solvent was removed in vacuum. The procedure was repeated, followed by addition of tetrahydrofuran (20 mL) and deuteroxide (2 mL) again. To this solution were added 1,1'-bis(diphenylphosphino)ferrocene-palladium(**II**)dichloride dichloromethane complex (0.11 g, 0.13 mmol) and N,N,N',N'-tetramethylethylenediamine (0.60 g, 5.14 mmol). After stirring for 10 min at room temperature, sodium borodeuteride (0.22 g, 5.14 mmol) was added in portion within 30 min. The mixture continued to be stirred at room temperature until the starting material disappeared completely. Then, the reaction was quenched with saturated NH_4Cl solution, and extracted with ethyl acetate. The organic phase was washed with brine, dried over Na_2SO_4, and concentrated. The residue was purified by chromatography on silica gel to give compound **1-3** with a deuterium content ⩾ 98% as a white solid (0.79 g, yield 55%).

Debenzylation of compound **1-3** (0.56 g, 1.0 mmol) with 1M BCl_3 dichloromethane solution (3.5 mmol, 3.5 mL) was performed according to the existing method[5] to afford compound **X1** as a white solid (0.15 g, yield 53%). ^{1}H NMR (500 MHz, DMSO-*d*$_6$) δ 8.01 – 7.79 (m, 3H), 6.88 (s, 1H), 6.09 (d, *J* = 6.4 Hz, 1H), 5.19 (d, *J* = 5.3 Hz, 1H), 4.91 (t, *J* = 5.8 Hz, 1H), 4.65 (t, *J* = 5.7 Hz, 1H), 4.08 – 4.04 (m, 1H), 3.98 – 3.93 (m, 1H), 3.67 – 3.61 (m, 1H), 3.54 – 3.48 (m, 1H). ^{13}C NMR (126 MHz, DMSO-*d*$_6$) δ 156.08, 148.32, 124.35, 117.82, 116.94, 111.16, 85.92, 79.04, 74.72, 70.56, 61.43. MS m/z = 293.0 $[M + 1]^+$.

Preparation of X2

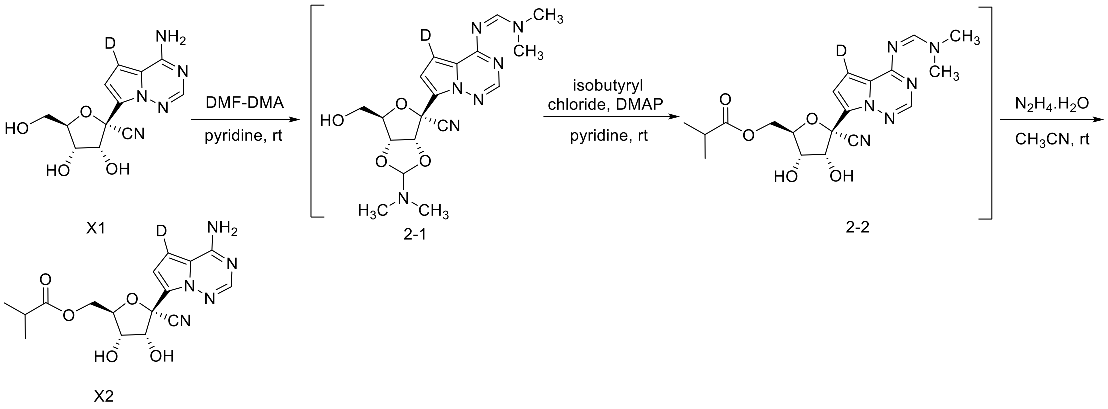

Compound **X1** (145 mg, 0.5 mmol) was added in pyridine (5 mL), and then the solvent was removed under vacuum. The process was repeated, followed by the addition of pyridine (5 mL) again. N,N-dimethylformamide dimethyl acetal (240 mg, 2.0 mmol) was added, and the mixture was stirred overnight at room temperature under nitrogen protection. The reaction mixture was concentrated to give intermediate **2-1**, which was taken directly to the following step.

The crude intermediate **2-1** was dissolved in pyridine (5 mL), and 4-dimethylaminopyridine (DMAP, 6 mg, 0.05 mmol) and isobutyryl chloride (80 mg, 0.75 mmol) were added at room temperature. The mixture was stirred for 3 h, then treated with methanol, and evaporated. The resulting oil residue was dissolved in acetonitrile (5 mL), followed by the addition of 85% hydrazine hydrate (176 mg, 3.0 mmol). The mixture was stirred overnight at room temperature, then poured into water, and extracted with ethyl acetate. The organic layer was separated, washed with brine, dried over Na_2SO_4, and concentrated. The reside was purified by chromatography on silica gel to give **X2** as a white solid (80 mg, yield 44%). ^1H NMR (600 MHz, DMSO-d_6) δ 8.01 – 7.79 (m, 3H), 6.80 (s, 1H), 6.32 (d, J = 6.0 Hz, 1H), 5.38 (d, J = 5.8 Hz, 1H), 4.69 (t, J = 5.4 Hz, 1H), 4.30 (dd, J = 12.1, 2.9 Hz, 1H), 4.25 – 4.21 (m, 1H), 4.17 (dd, J = 12.1, 5.3 Hz, 1H), 3.95 (q, J = 5.9 Hz, 1H), 2.54 – 2.51 (m, 1H), 1.06 (d, J = 2.9 Hz, 3H), 1.05 (d, J = 2.9 Hz, 3H). ^{13}C NMR (126 MHz, DMSO-d6) δ 175.98, 155.68, 148.02, 123.61, 117.02, 116.62, 110.24, 81.40, 79.12, 74.11, 70.27, 63.03, 33.25, 18.81, 18.73. MS m/z = 363.0 $[M + 1]^+$.

Preparation of X3

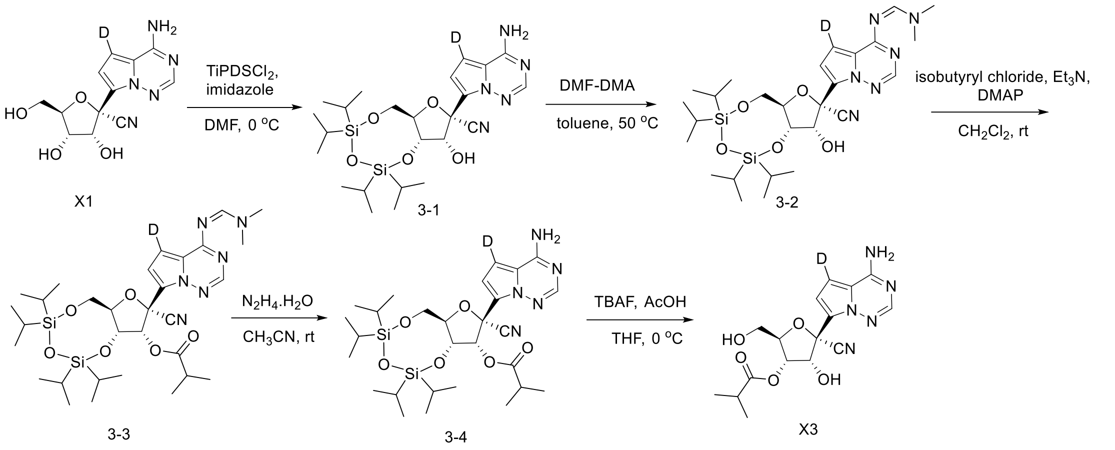

To a solution of compound **X1** (879 mg, 3.01 mmol) and imidazole (819 mg, 12.03 mmol) in DMF (15 mL), 1,3-dichloro-1,1,3,3-tetraisopropyldisiloxane (1.32 g, 4.21 mmol) was added dropwise under ice bath. After the addition, the mixture was stirred at room temperature for 4 h, then poured into water (100 mL), and extracted with ethyl acetate. The organic layer was separated, washed with brine, dried over Na_2SO_4, and concentrated. The residue was added in petroleum ether, and stirred overnight. The resulting solid was collected by filtration and dried to give compound **3-1** as a white solid (1.29 g, yield 80%). ^{1}H NMR (600 MHz, DMSO-d_6) δ 8.04 – 7.78 (m, 3H), 6.79 (s, 1H), 6.46 (d, J = 5.7 Hz, 1H), 4.56 (t, J = 5.1 Hz, 1H), 4.24 – 4.08 (m, 3H), 3.91 (d, J = 12.0 Hz, 1H), 1.08 – 0.86 (m, 28H).

Compound **3-1** (1.10 g, 2.06 mmol) was added in toluene (20 mL) followed by the addition of N-dimethylformamide dimethyl acetal (370 mg, 3.11 mmol). The mixture was stirred at 45 °C for about 30 min. The solvent was removed under vacuum to give compound **3-2** as a white foam (1.12g, yield 92%).

To a solution of compound **3-2** (614 mg, 1.04 mmol) in dichloromethane (10 mL) were successively added triethylamine (210 mg, 2.08 mmol), isobutyryl chloride (166 mg, 1.56 mmol) and DMAP (127 mg, 1.04 mmol). The mixture was stirred at room temperature for 1 h, and then treated with saturated sodium bicarbonate solution and dichloromethane. The organic layer was separated, dried over Na_2SO_4, concentrated, and purified by chromatography on silica gel to give compound **3-3** as a white foam (494 mg, yield 72%).

Compound **3-3** (350 mg, 0.53 mmol) was added in acetonitrile (8 mL) followed by the addition of 85% hydrazine hydrate (125 mg, 2.12 mmol) at room temperature. The mixture was stirred for about 30 min, then poured into water, and extracted with ethyl acetate. The organic layer was separated, washed with 1M hydrochloric acid, saturated sodium bicarbonate and brine, then dried over Na_2SO_4, and evaporated to give compound **3-4** as a white solid (289 mg, yield 90%).

To a solution of compound **3-4** (289 mg, 0.48 mmol) and acetic acid (7 mg, 0.12 mmol) in tetrahydrofuran (10 mL), 1 M solution of tetrabutylammonium fluoride in THF (0.48 mL, 0.48 mmol) was added in an ice bath. The mixture was stirred for 2–3 h, then poured into

water, and extracted with isopropyl acetate. The organic layer was separated, washed with saturated sodium bicarbonate and brine, then dried over Na_2SO_4 and evaporated. The crude product was slurried in n-heptane/isopropanol, and the resulting precipitate was filtrated to give compound **X3** as a white solid (121 mg, yield 70%). ^{1}H NMR (600 MHz, DMSO-*d₆*) δ 8.06 – 7.85 (m, 3H), 6.88 (s, 1H), 6.41 (d, *J* = 6.5 Hz, 1H), 5.21 (dd, *J* = 5.7, 3.3 Hz, 1H), 5.08 – 5.04 (m, 1H), 4.99 (t, *J* = 6.0 Hz, 1H), 4.26 (q, *J* = 3.7 Hz, 1H), 3.64 – 3.52 (m, 2H), 2.67 – 2.58 (m, 1H), 1.17 (d, *J* = 7.0 Hz, 3H), 1.15 (d, *J* = 7.0 Hz, 3H). ^{13}C NMR (126 MHz, DMSO-d_6) δ 175.92, 156.13, 148.45, 123.24, 117.50, 117.27, 111.60, 84.59, 78.30, 73.14, 72.69, 61.25, 33.81, 19.18, 19.08. MS m/z = 363.0 [M + 1]$^+$. MS m/z = 363.0 [M + 1]$^+$.

Preparation of X4

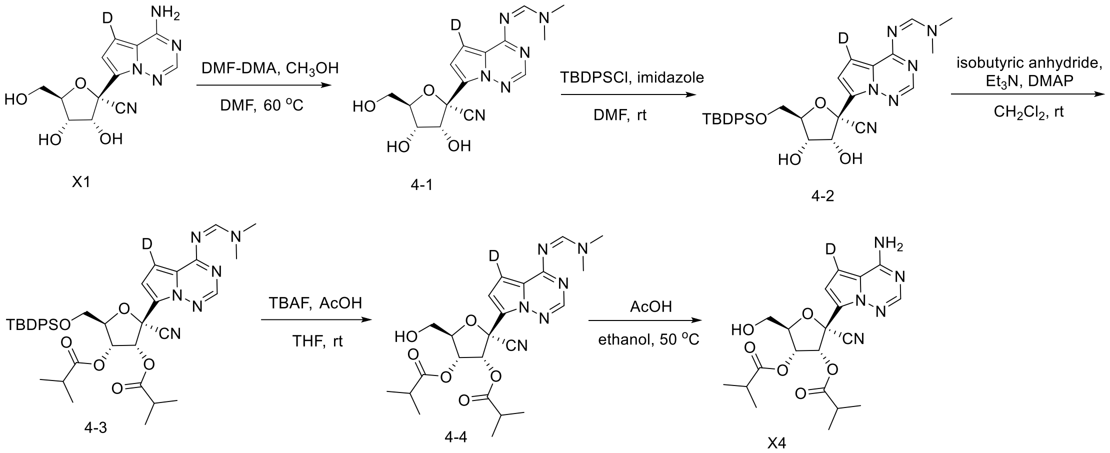

To a solution of **X1** (0.58 g, 2.0 mmol) in DMF (10 mL) was added N,N-dimethylformamide dimethyl acetal (1.43 g, 12.0 mmol) at room temperature. The mixture was stirred at 60 °C for 2 h, then cooled to room temperature, and an equivalent volume of methanol was added. The solvent was evaporated under vacuum to give an oily residue which was treated with isopropyl alcohol. The resulting precipitate was collected by filtration and dried to give compound **4-1** as an off-white solid (0.55 g, 80% yield).

To a mixture of **4-1** (1.51 g, 4.34 mmol) and imidazole (1.18 g, 17.37 mmol) in DMF (15 mL), tert-butyldiphenylchlorosilane (2.39 g, 8.69 mmol) was added in an ice bath. The mixture was stirred at room temperature for 5 h, then poured into water, and extracted with ethyl acetate. The organic phase was separated, washed with diluted hydrochloric acid (0.5 M), saturated $NaHCO_3$ aqueous solution and brine, dried over Na_2SO_4, and concentrated. The residue was subjected to chromatography on silica gel to give compound **4-2** as a foam solid (2.05 g, yield 81%).

To a solution of **4-2** (2.05 g, 3.50 mmol) in CH_2Cl_2 (30 mL), triethylamine (1.24 g, 12.26 mmol), DMAP (0.21 g, 1.75 mmol) and isobutyric anhydride (1.39 g, 8.76 mmol) were added successively at room temperature under nitrogen protection. The mixture was stirred for 1 h, and concentrated. The residue was partitioned between ethyl acetate and

water. The organic layer was washed with 0.5 M diluted hydrochloric acid, saturated $NaHCO_3$ aqueous solution and brine, dried over Na_2SO_4, and concentrated. The crude product **4-3** was used for the next step without purification.

To a solution of the crude product **4-3** in THF (30 mL), acetic acid (0.11 g, 1.75 mmol) and 1M tetrabutylammonium fluoride THF solution (3.5 mL, 3.5 mmol) were added at room temperature. The mixture was stirred for 2 h, and partitioned between ethyl acetate and water. The organic layer was washed with saturated $NaHCO_3$ aqueous solution and brine, dried over Na_2SO_4, and concentrated. The crude product **4-4** was used for the next step without purification.

The crude intermediate **4-4** was added in ethanol (20 mL), followed by the addition of acetic acid (4.2 g, 70 mmol) at room temperature. The mixture was stirred at 50 °C overnight, and concentrated. The residue was partitioned between ethyl acetate and water. The organic layer was washed with saturated $NaHCO_3$ aqueous solution and brine, dried over Na_2SO_4, and concentrated. The reside was purified by chromatography on silica gel to give compound **X4** as a gummy solid (0.85 g, yield 56% over three steps). ^{1}H NMR (500 MHz, DMSO-d_6) δ 8.15 – 7.89 (m, 3H), 6.80 (s, 1H), 6.01 (d, J = 5.7 Hz, 1H), 5.44 (dd, J = 5.7, 3.1 Hz, 1H), 5.18 (dd, J = 6.1, 5.2 Hz, 1H), 4.41 (q, J = 3.4 Hz, 1H), 3.71 – 3.60 (m, 2H), 2.69 – 2.54 (m, 2H), 1.19 (d, J = 7.0 Hz, 3H), 1.16 (d, J = 7.0 Hz, 3H), 1.11 (d, J = 2.4 Hz, 3H), 1.09 (d, J = 2.4 Hz, 3H). MS m/z = 433.2 $[M + 1]^+$.

Preparation of X5

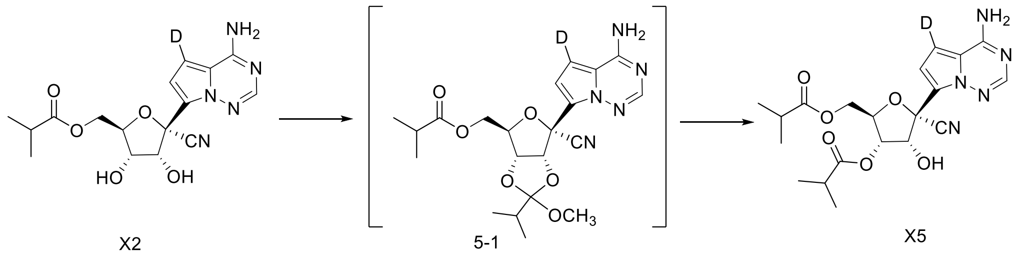

X2 (0.18 g, 0.5 mmol) and 1,1,1-trimethoxy-2-methylpropane (0.37 g, 2.5 mmol) were added in acetic acid (3 mL) at room temperature. The mixture was stirred at 50 °C until the starting material disappeared. Then, the solvent was removed in vacuum to give an oil residue which was dissolved in tetrahydrofuran (6 mL). To this solution, 1M hydrochloric acid (0.5 mL) was added. After stirring for 1 h, the mixture was neutralized with saturated $NaHCO_3$ aqueous solution to a pH of ~7, and extracted with ethyl acetate. The organic phase was separated, washed with brine, dried over Na_2SO_4, and concentrated. The residue was crystallized in isopropanol/H_2O to give **X5** as a white solid (0.18 g, yield 82%). ^{1}H NMR (600 MHz, DMSO-d_6) δ 8.05 – 7.83 (m, 3H), 6.85 (s, 1H), 6.56 (d, J = 6.5 Hz, 1H), 5.16 (dd, J = 5.6, 4.1 Hz, 1H), 5.09 (t, J = 6.1 Hz, 1H), 4.46 (q, J = 4.3 Hz, 1H), 4.27 (dd, J = 12.2, 4.0 Hz, 1H), 4.23 (dd, J = 12.2, 4.8 Hz, 1H), 2.67 – 2.58 (m, 1H), 2.55 – 2.49 (m, 1H), 1.16 (d, J = 7.0 Hz, 3H), 1.15 (d, J = 7.0 Hz, 3H), 1.05 (d, J = 7.0 Hz, 3H), 1.03 (d, J

= 7.2 Hz, 3H). MS m/z = 433.2 [M + 1]$^+$.

Preparation of X6

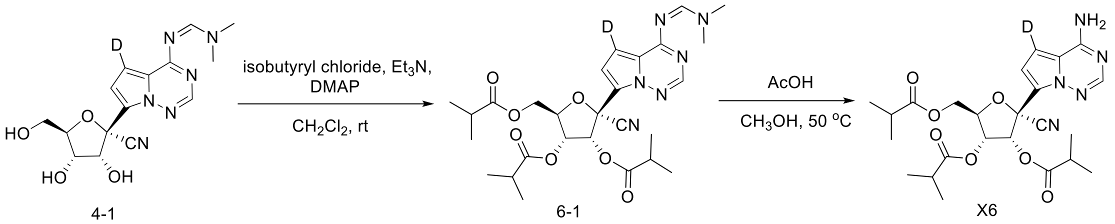

Compound **4-1** (0.37 g, 1.0 mmol), triethylamine (0.81 g, 8.0 mmol) and DMAP (0.37 g, 3.0 mmol) were suspended in dichloromethane (10 mL), and to this solution isobutyryl chloride (0.43 g, 4.0 mmol) was slowly added at room temperature. The mixture was stirred until the starting material completely disappeared. The solvent was removed under vacuum, and the residue was partitioned between water and ethyl acetate. The organic layer was separated, washed successively with 1M hydrochloric acid, saturated sodium bicarbonate and brine, then dried over Na_2SO_4, and evaporated to give the crude product **6-1** as an oil, which was taken directly to the following step without further purification.

The crude intermediate **6-1** was dissolved in ethanol (10 mL), followed by the addition of acetic acid (1.2 g, 20.0 mmol) at room temperature. The mixture was stirred at 50 °C overnight, and concentrated. The resulting residue was purified by chromatography on silica gel to afford compound **X6** as a white foam solid (0.38 g, 75% yield over two steps). ^{1}H NMR (500 MHz, DMSO-d_6) δ 8.04 (br, 1H), 7.98 (br, 1H), 7.94 (s, 1H), 6.77 (s, 1H), 6.09 (d, *J* = 5.7 Hz, 1H), 5.45 (dd, *J* = 5.8, 3.7 Hz, 1H), 4.64 (q, *J* = 3.7 Hz, 1H), 4.34 (dd, *J* = 12.4, 3.3 Hz, 1H), 4.29 (dd, *J* = 12.4, 4.1 Hz, 1H), 2.69 – 2.56 (m, 2H), 2.51 – 2.46 (m, 1H), 1.19 (d, *J* = 7.1 Hz, 3H), 1.16 (d, *J* = 6.9 Hz, 3H), 1.14 – 1.10 (m, 6H), 1.06 (d, *J* = 7.0 Hz, 3H), 1.03 (d, *J* = 7.0 Hz, 3H). ^{13}C NMR (126 MHz, DMSO-d_6) δ 175.53, 174.90, 174.13, 155.58, 148.12, 120.98, 117.17, 115.44, 110.30, 81.25, 75.81, 72.05, 70.30, 62.46, 33.20, 33.16, 33.09, 18.55, 18.46, 18.40, 18.35, 18.33, 18.18. MS m/z = 503.0 [M + 1]$^+$.

Preparation of X6 hydrobromide (VV116)

To a solution of **X6** (12.0 g, 23.9 mmol) in acetonitrile (100 mL), 40 wt.% hydrobromic acid in H_2O (4.37 g, 21.6 mmol) was slowly added in an ice bath. After the addition, the mixture was allowed to warm to room temperature and continued to be stirred for 30 mins. The solvent was removed in vacuum, followed by the addition of methyl tert-butyl ether (150 mL). The mixture was stirred for 30 mins at room temperature, then stirred for 2 h at 55 °C, and cooled to room temperature. The solid was collected by filtration, and dried to give a white solid (10.8 g, yield 78%). ^{1}H-NMR (600 MHz, $CDCl_3$): *δ* 13.05 (s, 1H), 9.73 (s, 1H), 9.46 (s, 1H), 8.00 (s, 1H), 7.04 (s, 1H), 6.02 (d, *J* = 5.8 Hz, 1H), 5.41 (dd, *J* = 5.8, 4.0 Hz, 1H), 4.65 (q, *J* = 4.0 Hz, 1H), 4.40 – 4.33 (m, 2H), 2.71 – 2.60 (m, 2H), 2.58 – 2.52 (m, 1H),

1.26 – 1.22 (m, 6H), 1.21 – 1.18 (m, 6H), 1.17 – 1.13 (m, 6H). ^{13}C-NMR (150 MHz, DMSO-d_6): δ 176.01, 175.35, 174.59, 150.65, 139.98, 125.81, 115.79, 115.43, 112.21, 82.22, 75.64, 73.33, 70.72, 62.99, 33.67, 33.62, 33.56, 19.11, 19.02, 18.92, 18.87, 18.85, 18.70. MS: *m/z* 503.1 $[M+1]^+$.

^{1}H NMR and ^{13}C NMR spectrum of X1–X6.

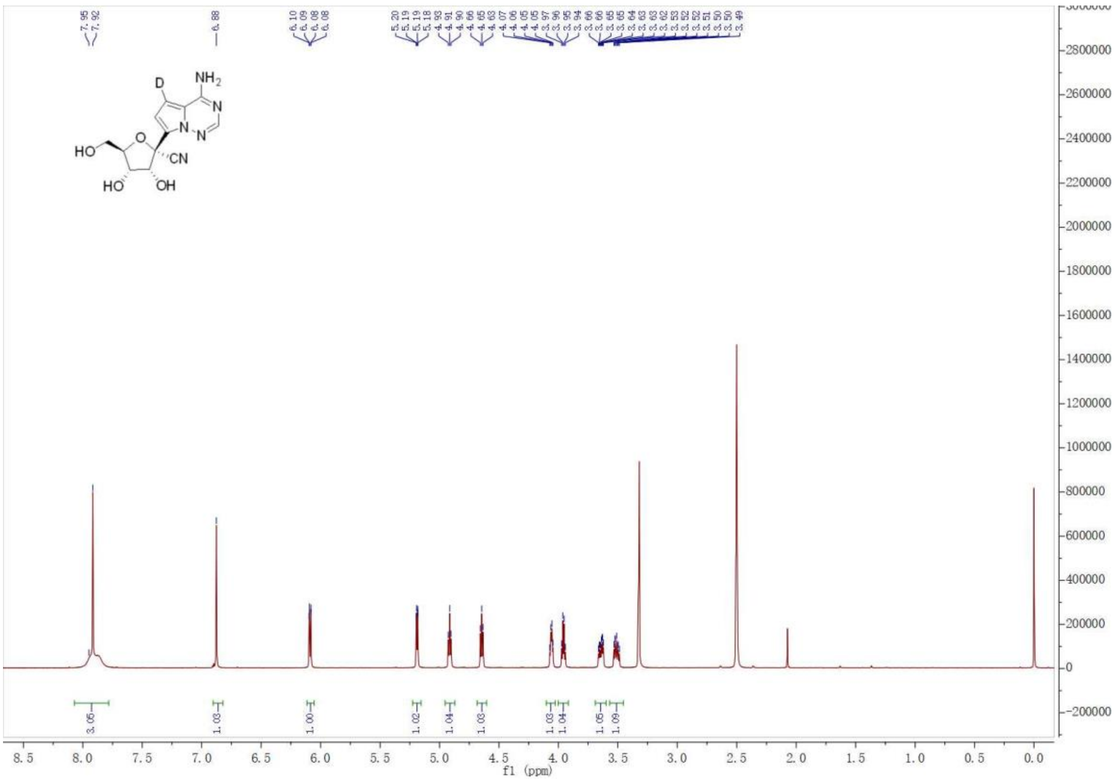

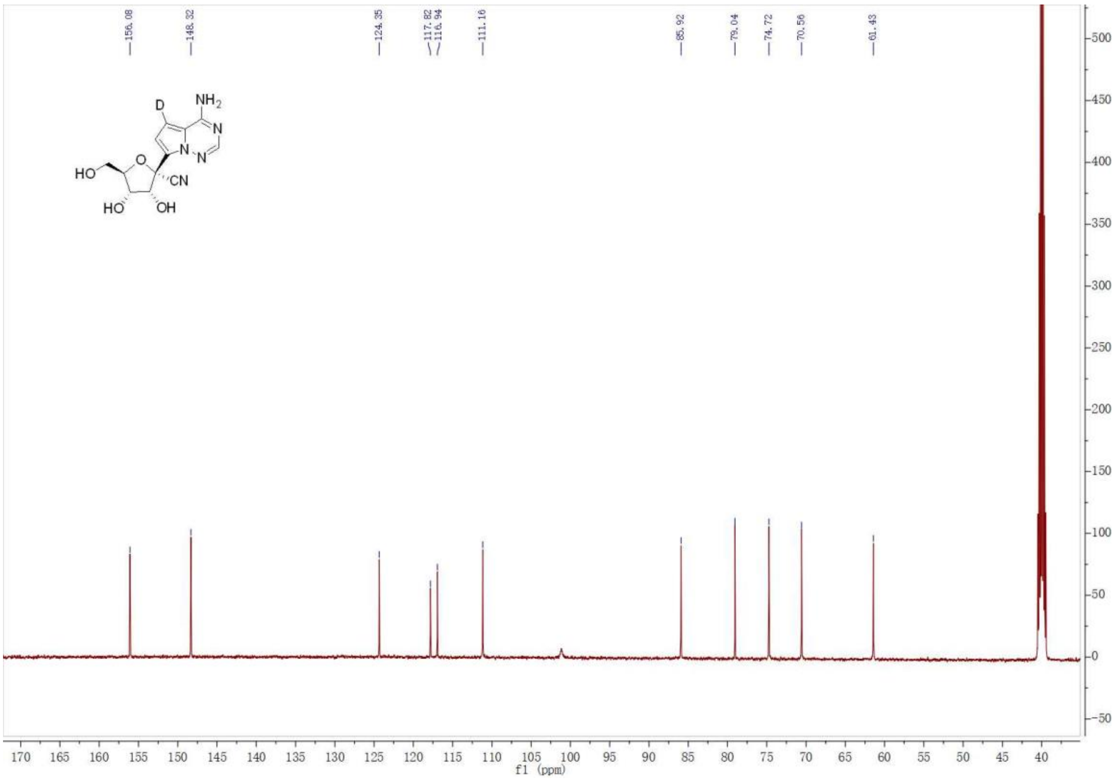

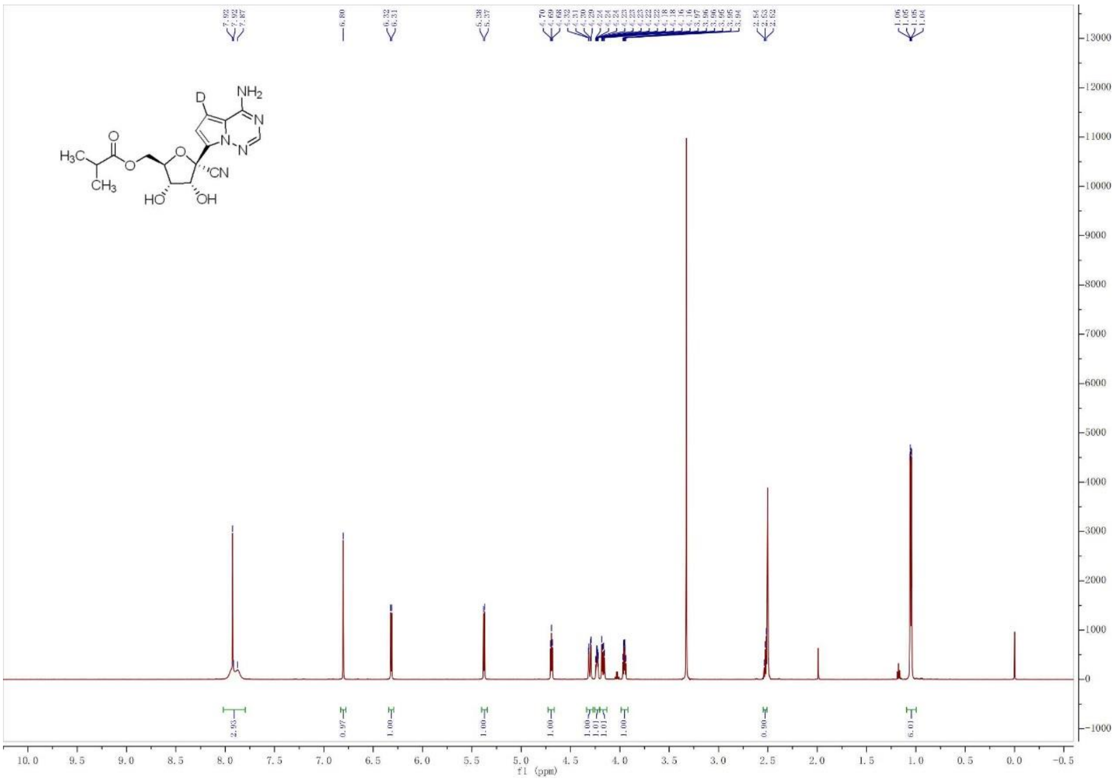

D
NH_2
H_3C
CH_3
O
N
CN
HO
OH
f1 (ppm)

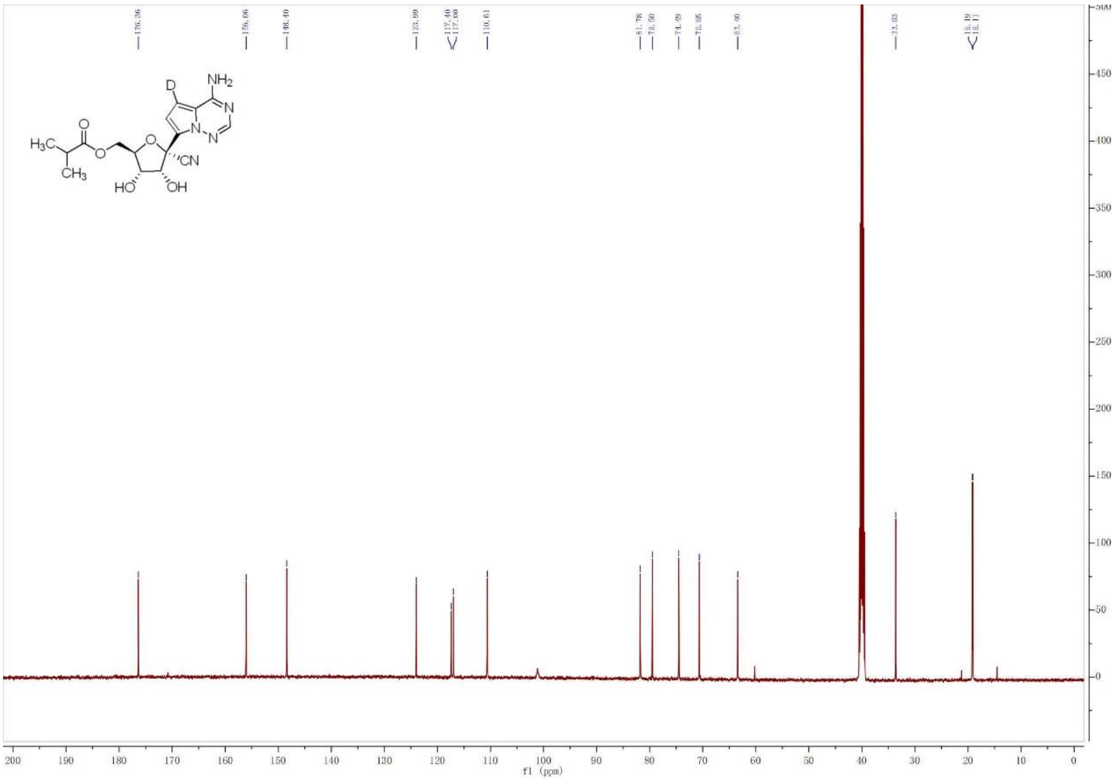

D
NH_2
H_3C
CH_3
O
N
CN
HO
OH
f1 (ppm)

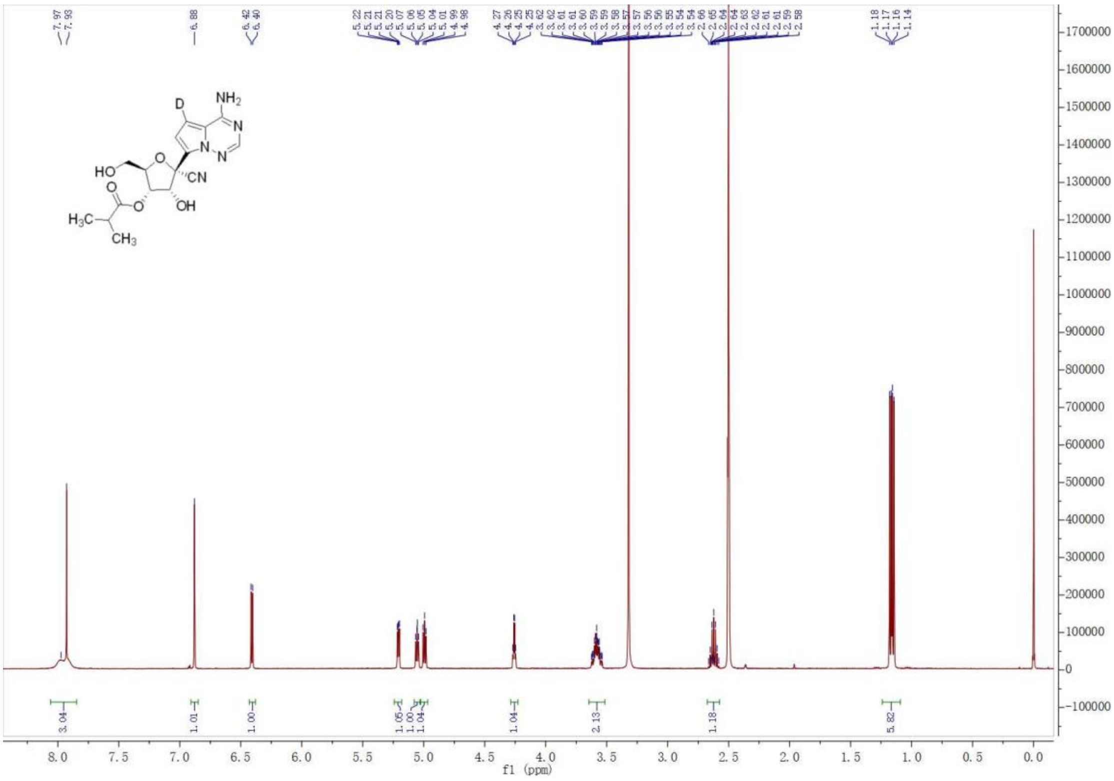

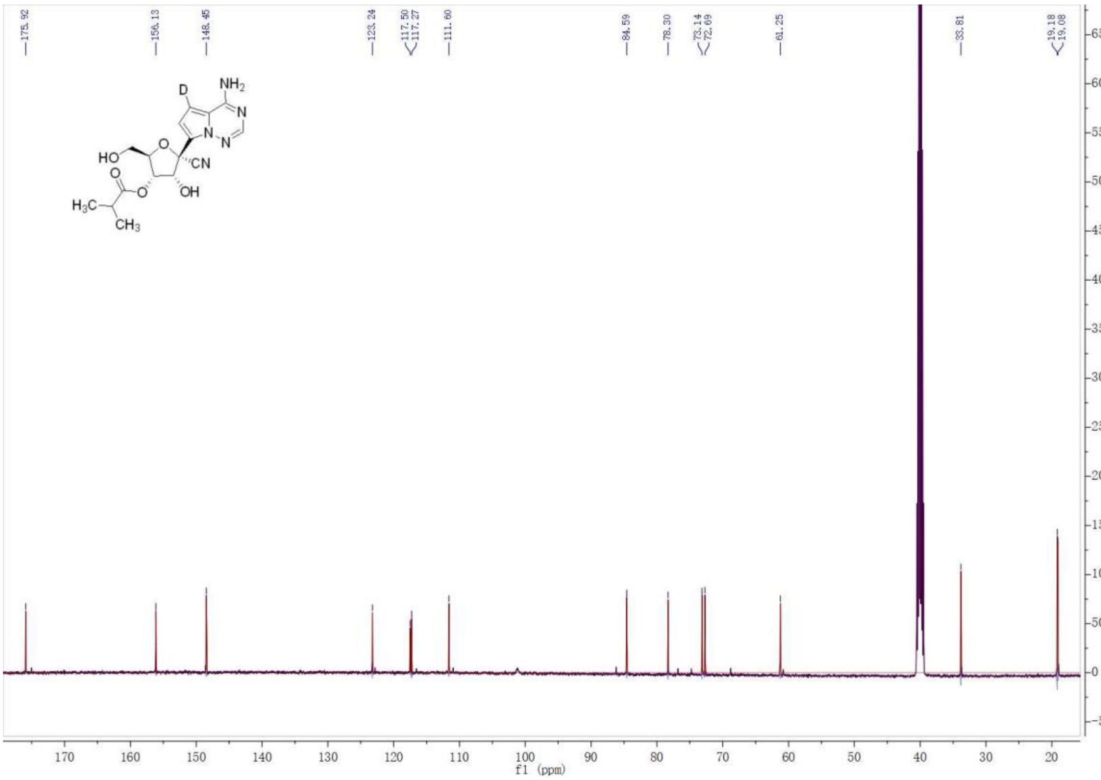

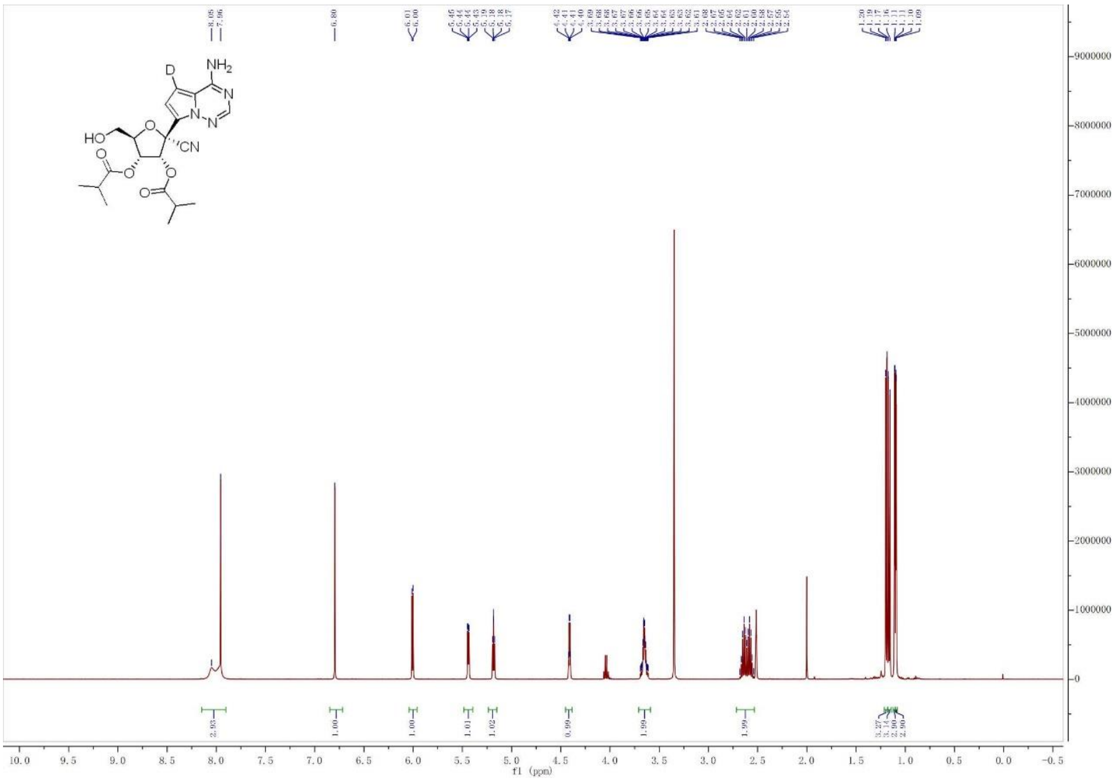
D
NH2
HO
CN
f1 (ppm)

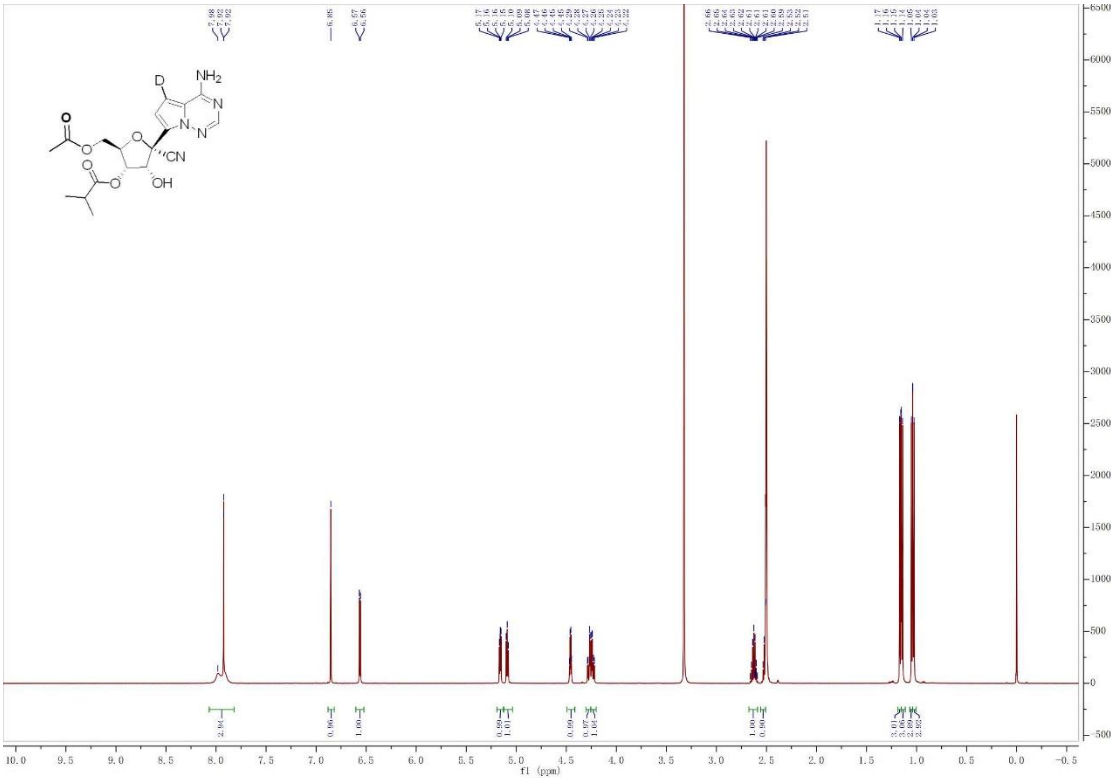
D
NH2
CN
OH
f1 (ppm)

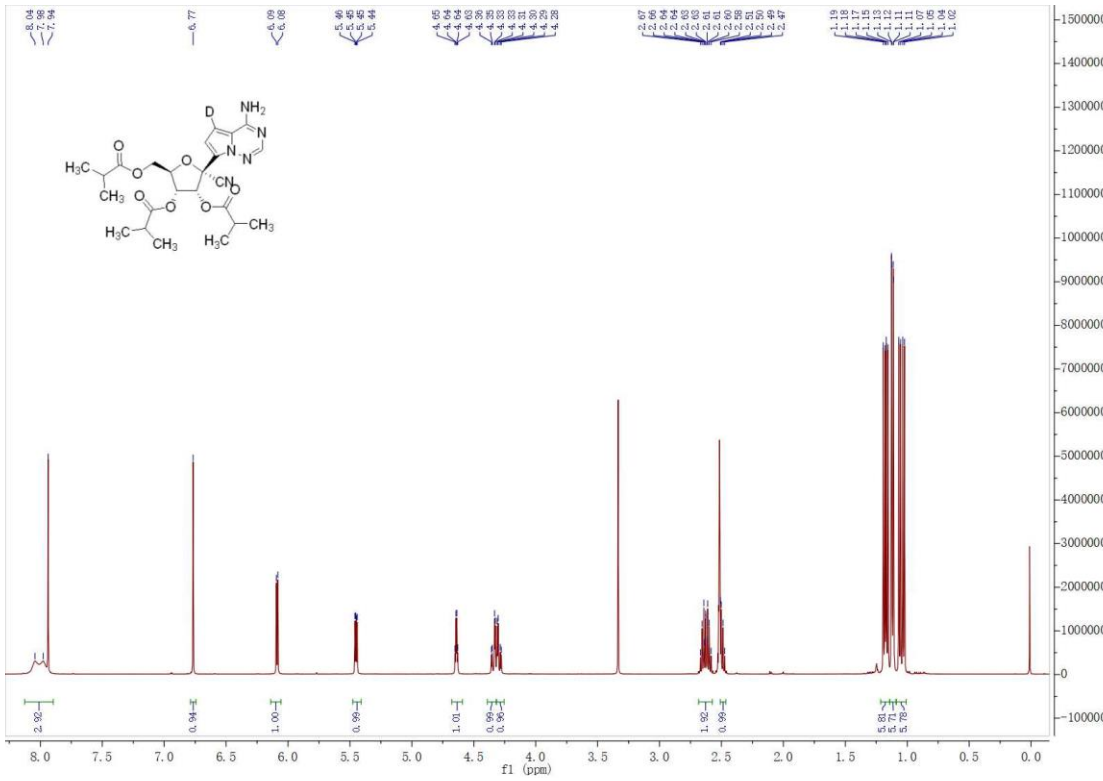
8.04
7.98
7.94
6.77
6.09
6.08
5.46
5.45
5.45
5.44
4.65
4.64
4.64
4.63
4.36
4.35
4.33
4.33
4.31
4.30
4.29
4.28
2.67
2.66
2.64
2.64
2.63
2.63
2.61
2.61
2.60
2.58
2.51
2.50
2.49
2.47
1.19
1.18
1.17
1.15
1.13
1.12
1.11
1.11
1.07
1.05
1.04
1.02
D
NH_2
N
N
N
O
O
O
O
O
O
O
CN
H_3C
CH_3
H_3C
CH_3
H_3C
CH_3
2.92
0.94
1.00
0.99
1.01
0.99
0.96
1.92
0.99
5.81
5.71
5.78
f1 (ppm)

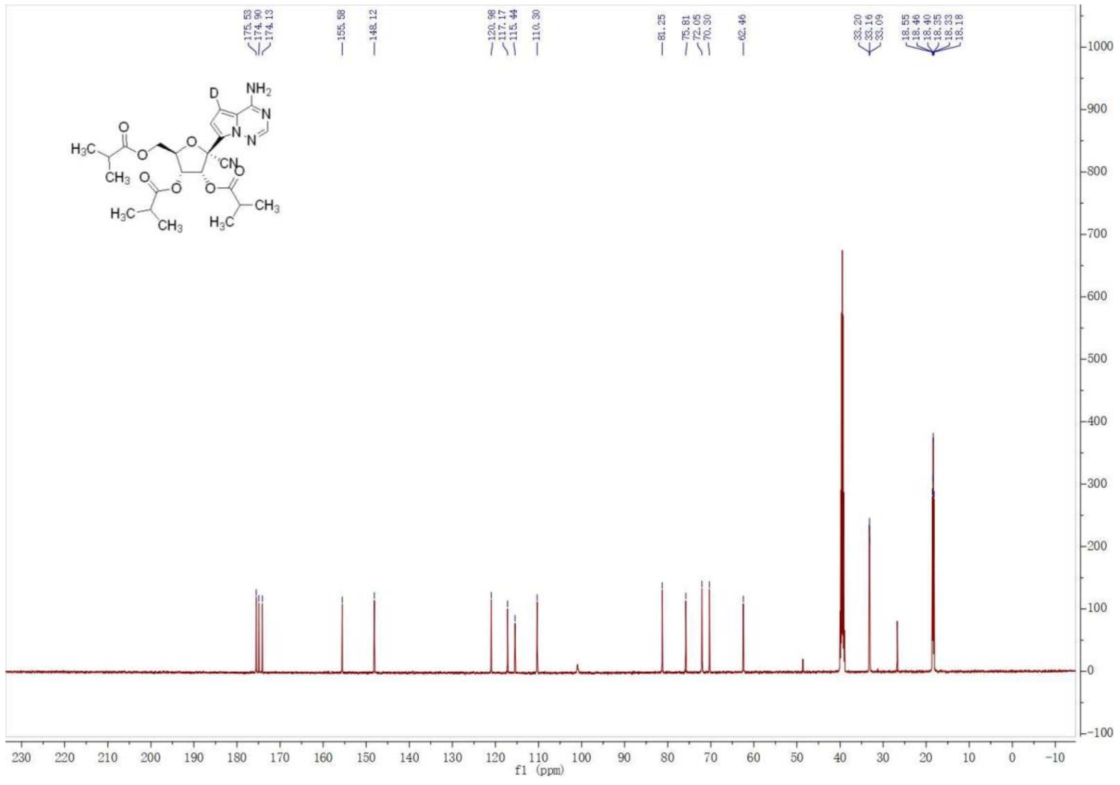
175.53
174.90
174.13
155.58
148.12
120.98
117.17
115.44
110.30
81.25
75.81
72.05
70.30
62.46
33.20
33.16
33.09
18.55
18.46
18.40
18.35
18.33
18.18
D
NH_2
N
N
N
O
O
O
O
O
O
O
CN
H_3C
CH_3
H_3C
CH_3
H_3C
CH_3
f1 (ppm)

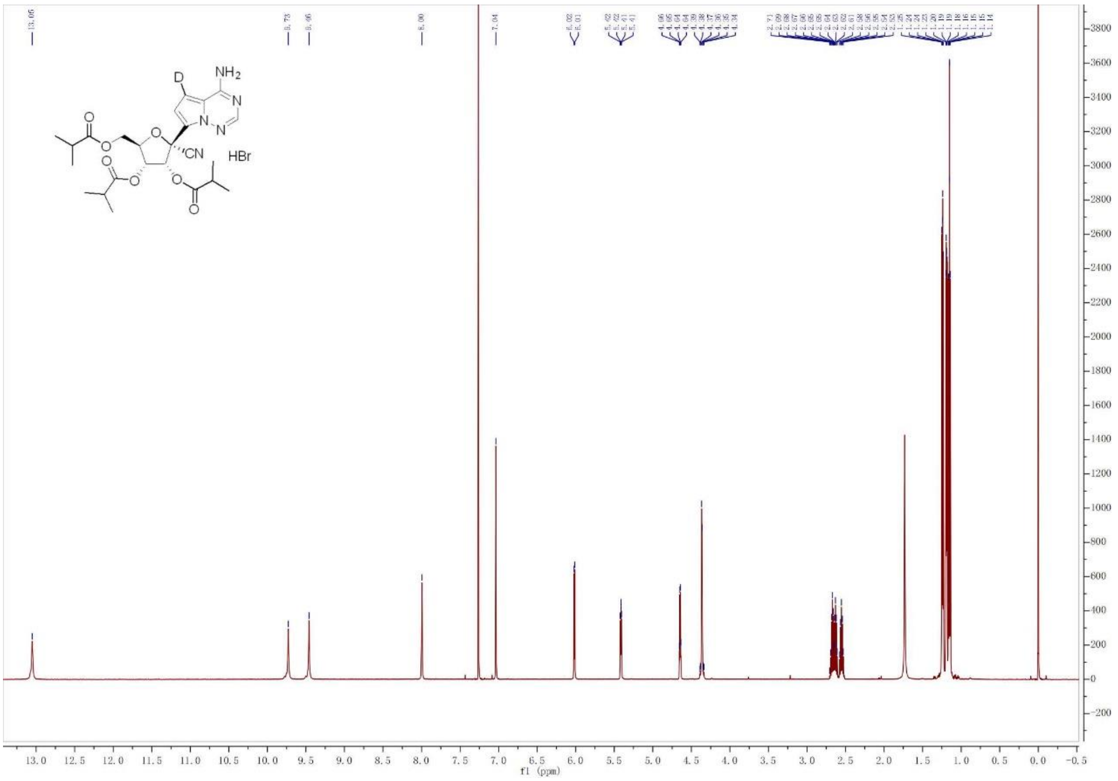

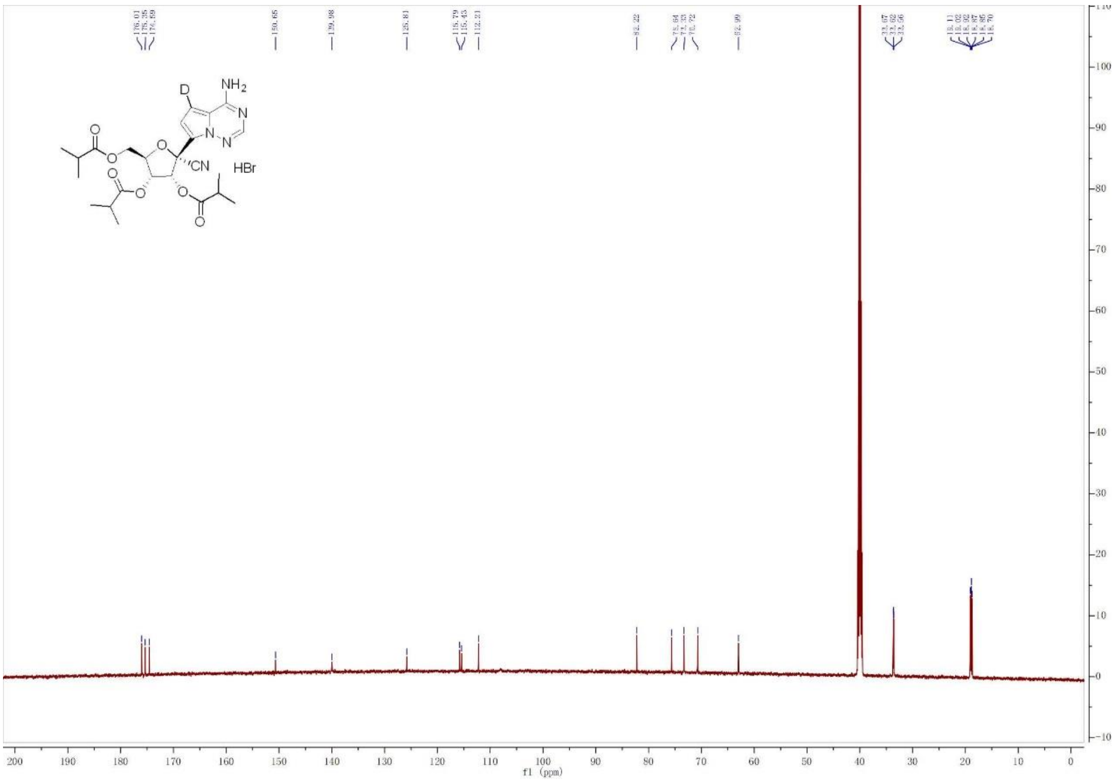

Reference

1 Wang, M. *et al.* Remdesivir and chloroquine effectively inhibit the recently emerged novel coronavirus (2019-nCoV) in vitro. *Cell Res.* **30**, 269-271, doi:10.1038/s41422-020-0282-0 (2020).

2 Metobo, S. E. *et al.* Practical synthesis of 1′-subst.ituted Tubercidin C-nucleoside analogs.

Tetrahedron Lett. **53**, 484-486, doi:https://doi.org/10.1016/j.tetlet.2011.11.055 (2012).

3 Clarke, M. O. N. H., Kim, C. U. & Lew, W. 2'-Fluoro substituted carba-nucleoside analogs for antiviral treatment. WO2012037038 (2012).

4 Wei, D. *et al.* Potency and pharmacokinetics of GS-441524 derivatives against SARS-CoV-2. *Bioorg. Med. Chem.* **46**, 116364, doi:10.1016/j.bmc.2021.116364 (2021).

5 Warren, T. K. *et al.* Therapeutic efficacy of the small molecule GS-5734 against Ebola virus in rhesus monkeys. *Nature* **531**, 381-385, doi:10.1038/nature17180 (2016).

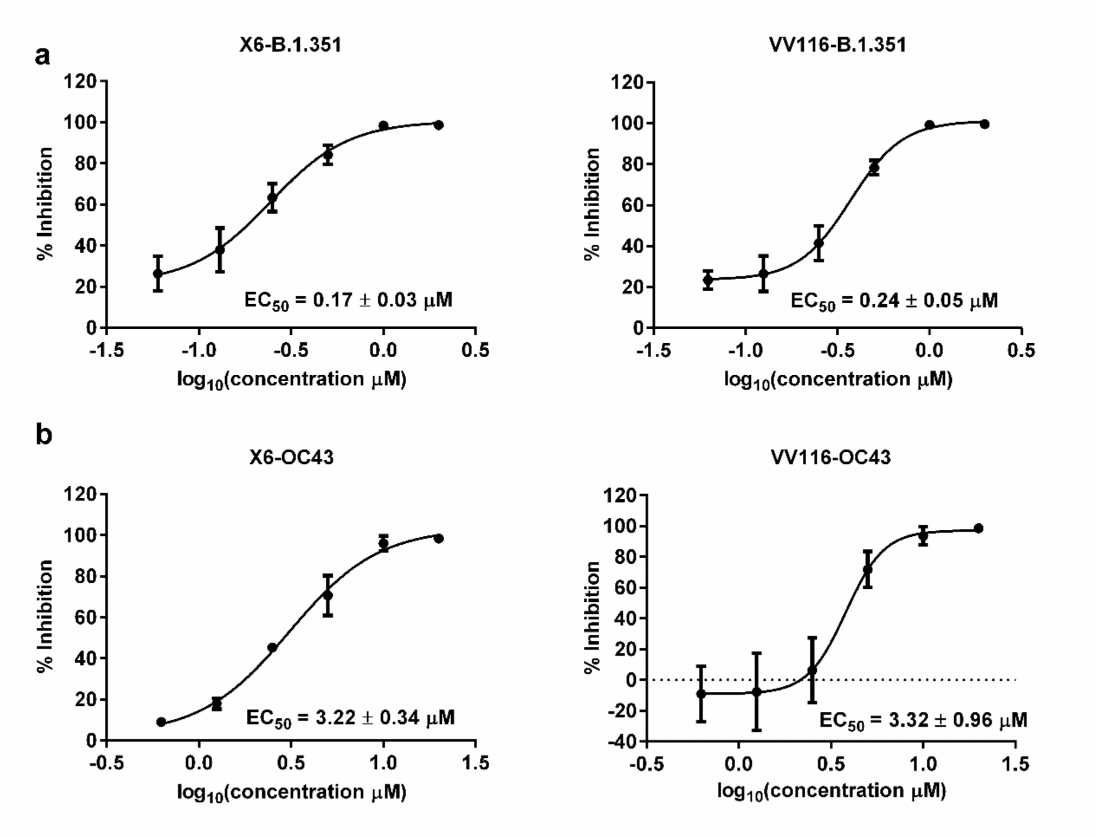

Supplementary information, Fig. S1: The effects of X6 and VV116 against SARS-CoV-2 B.1.351 and OC43-CoV in vitro.

a The effects of X6 and VV116 against SARS-CoV-2 B.1.351 in Vero E6 cells. **b** The effects of X6 and VV116 against OC43-CoV in RD cells.

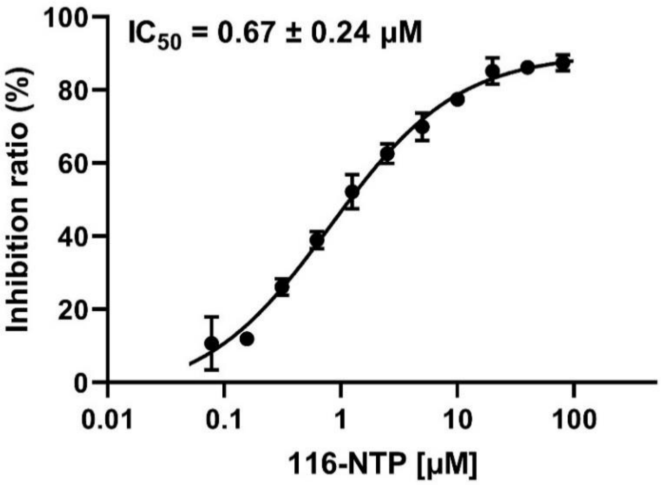

Supplementary information, Fig. S2: The inhibitory activity of the nucleoside triphosphate of VV116 (116-NTP) against the SARS-CoV-2 RNA-dependent RNA polymerase.

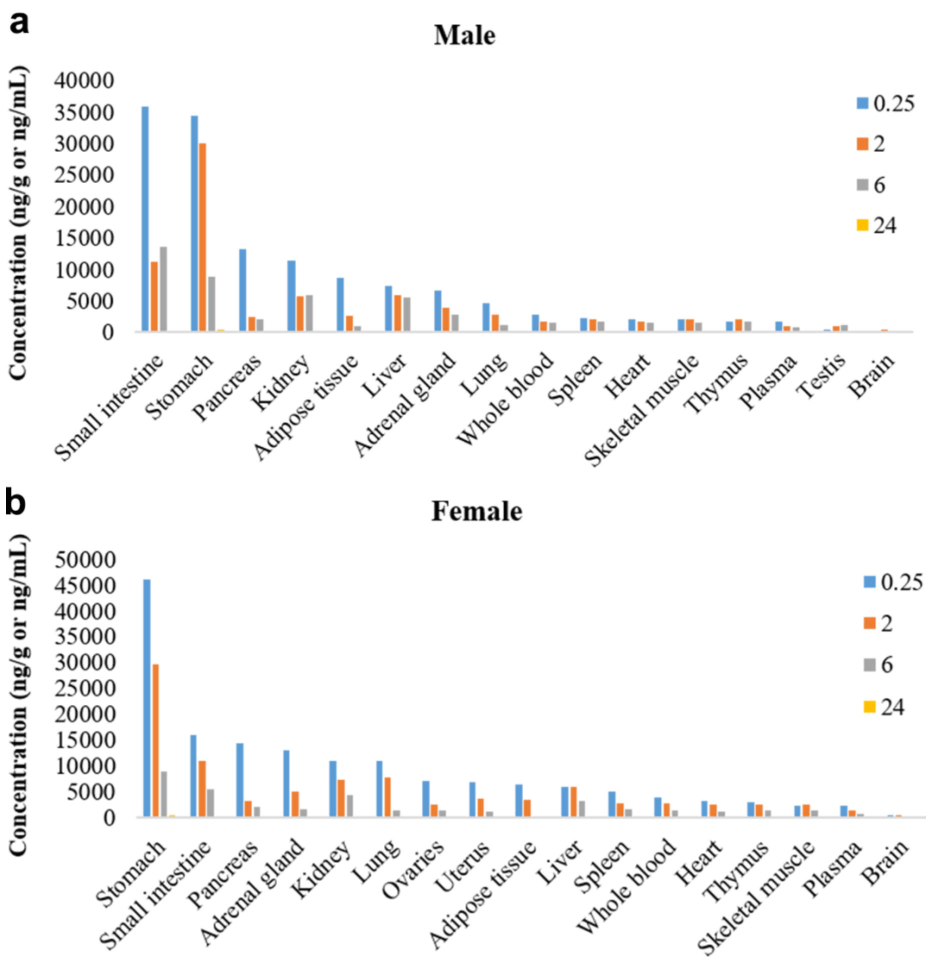

Supplementary information, Fig. S3: Tissue distribution of the nucleoside metabolite X1 in male (a) and female (b) rats following a single oral administration of VV116 at 30 mg/kg.

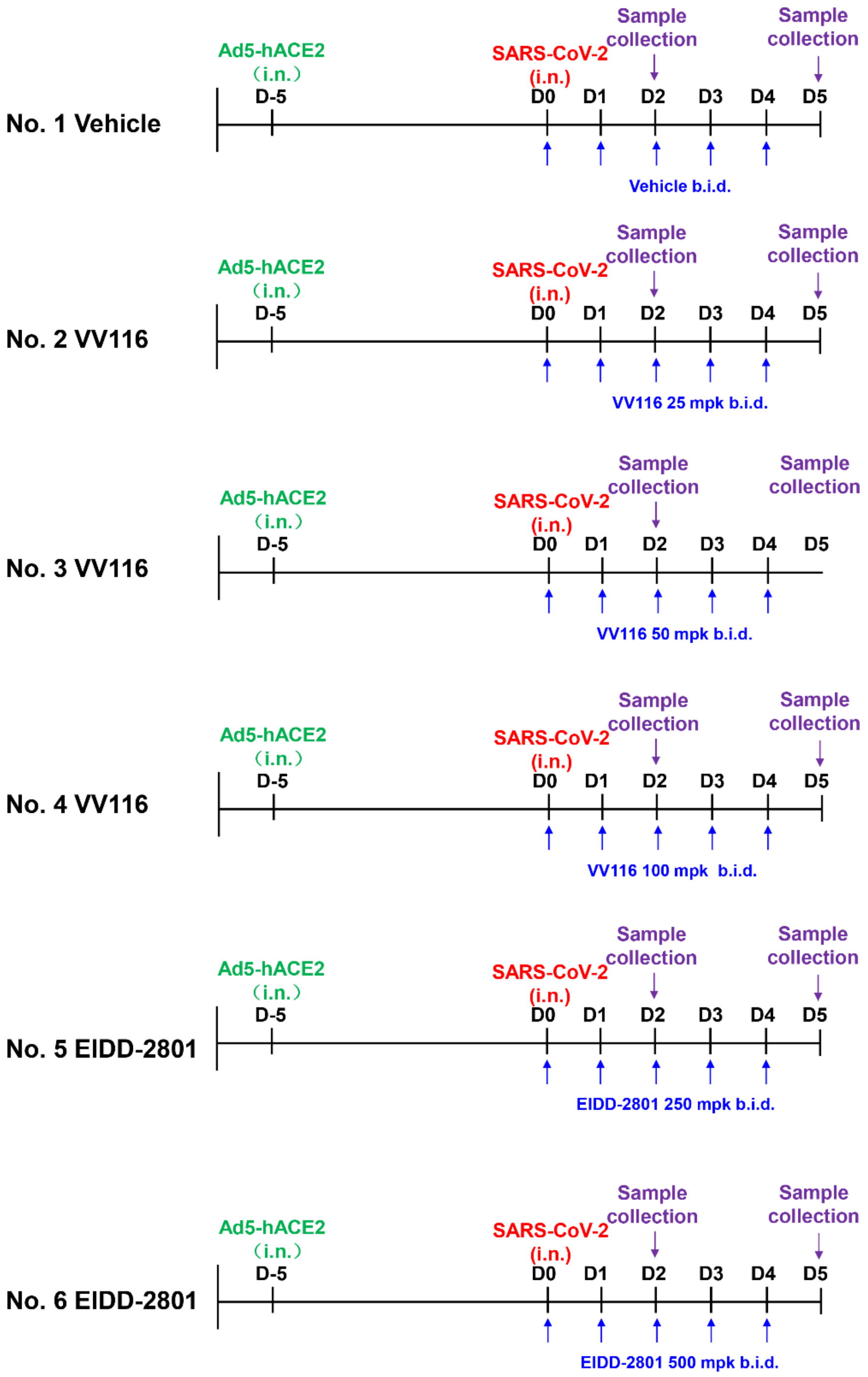

Supplementary information, Fig. S4: The design of the in vivo anti-SARS-CoV-2 efficacy study of VV116 in Ad5-hACE2 mice. 6-to-8-week-old female BALB/c mice were intranasally infected with 50 μL 5×10^9 PFU/ml of Ad5-hACE2 (day -5). At day 0, mice were infected with 1×10^5 PFU of SARS-CoV-2 and treated with vehicle orally (Vehicle, n=10), VV116 25 mg/kg orally b.i.d. (VV116, 25 mpk, n=10), VV116 50 mg/kg orally b.i.d. (VV116, 50 mpk, n=10), VV116 100 mg/kg orally b.i.d. (VV116, 100 mpk, n=10), EIDD-2801 250 mg/kg orally b.i.d. (EIDD-2801, 250 mpk, n=10), or EIDD-2801 500 mg/kg orally b.i.d. (EIDD-2801, 500 mpk, n=10) from day 0 to day 4. Mice were sacrificed at day 2 and day 5.

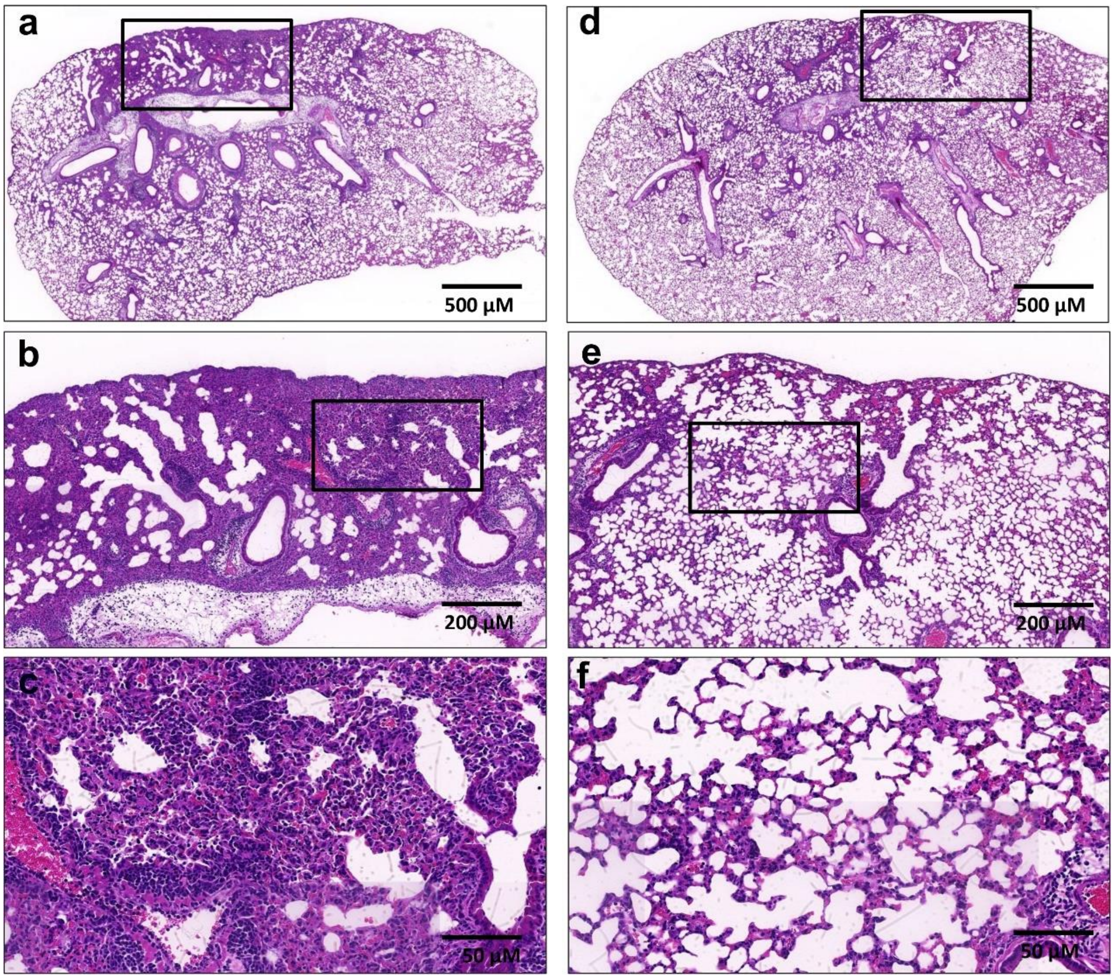

Supplementary information, Fig. S5: Histopathology of the lungs of the vehicle-treated and 100 mg/kg VV116-treated mice.

a, b, c Representative H&E images of lung sections of vehicle-treated mice, moderate interstitial pneumonia with thickened alveolar septa, infiltration of lymphocytes and necrotic debris. **b** the magnification of the black box shown in **a**; **c** the magnification of the black box shown in **b**. **d, e, f** Representative H&E images of lung sections of 100 mg/kg V116-treated mice, mild interstitial pneumonia with a small aera of inflammation and a low-grade lesion. **e** the magnification of the black box shown in **d**; **f** the magnification of the black box shown in **e**.

Supplementary information, Table S1: A list of nucleoside and nucleotide analogues which displayed no anti-SARS-CoV-2 activity at the concentration of 5.0 μM in Vero E6 cells

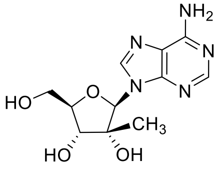	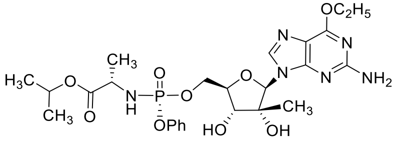	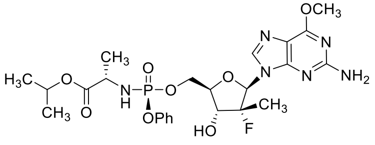	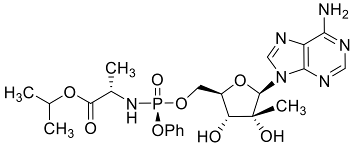	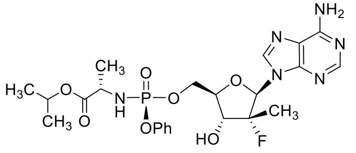
a1	a2	a3 (PSI-353661)	a4	a5
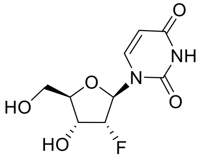	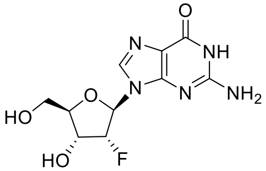	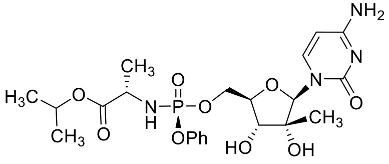	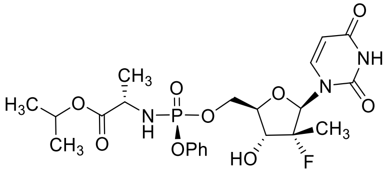	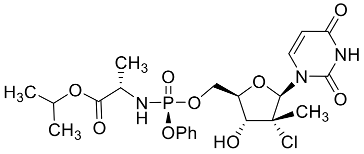
a6	a7	a8	a9 (sofosbuvir)	a10
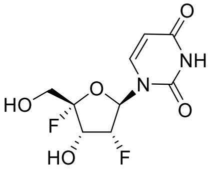	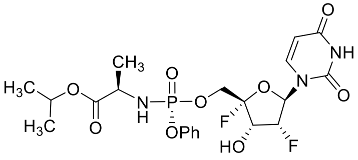	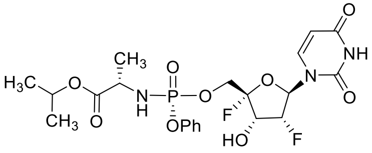	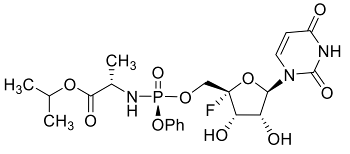	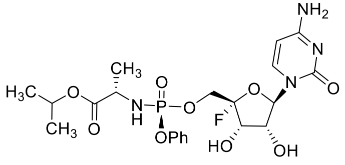
a11	a12	a13	a14	a15

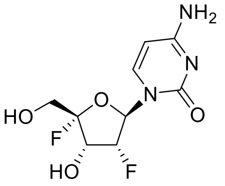	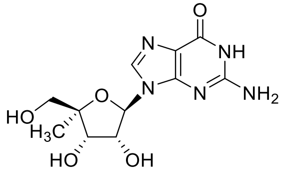	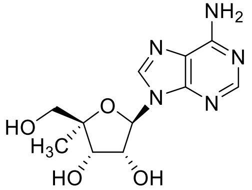	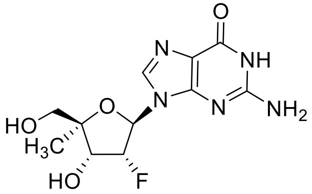	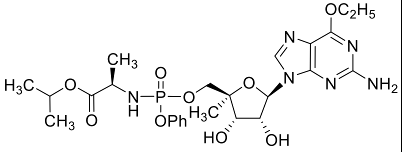
a16	a17	a18	a19	a20
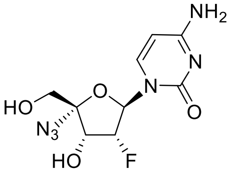	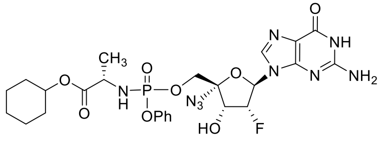	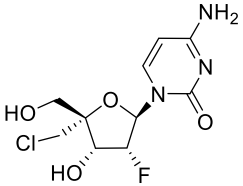	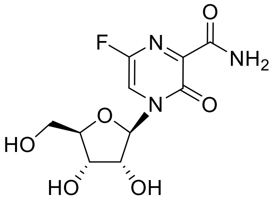	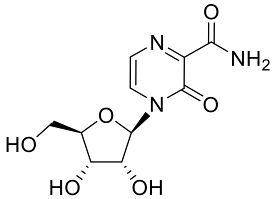
a21	a22	a23 (AL-8716)	a24	a25 (T-1106)
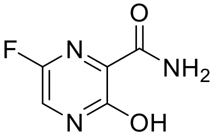	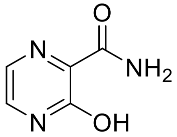	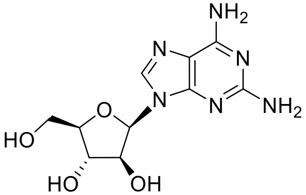	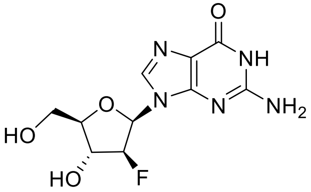	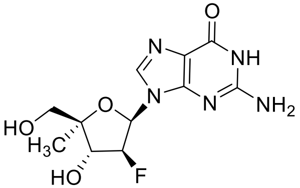
a26 (favipiravir)	a27 (T-1105)	a28	a29	a30
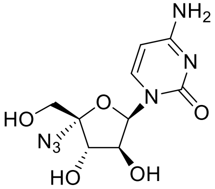	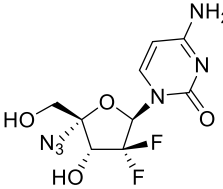	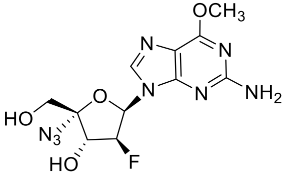	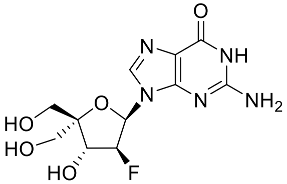	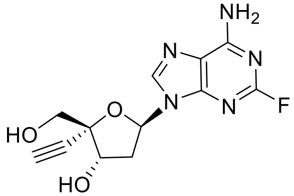
a31	a32	a33	a34	a35 (EFdA)

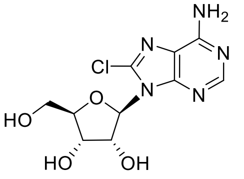	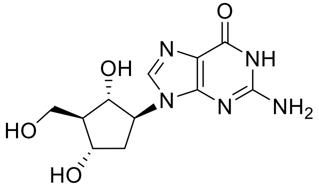	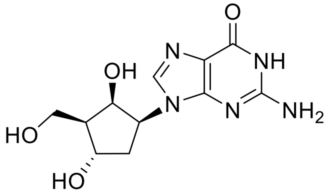	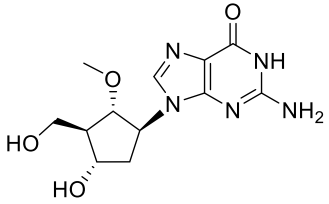	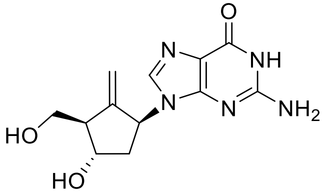
a36	a37	a38	a39	a40 (entecavir)
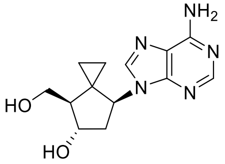	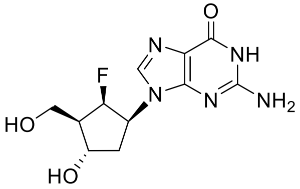	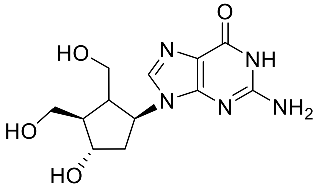		
a41	a42	a43		

Supplementary information, Table S2: Antiviral activity and cytotoxicity of GS-441524 derivatives against SARS-CoV-2 in Vero E6 cells

Compound	Structure	EC_{50} (μM)	CC_{50} (μM)
GS-441524	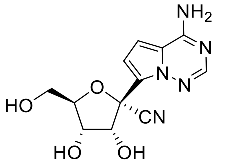	0.59 ± 0.1	> 500
A1	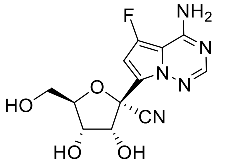	3.30	NT
X1	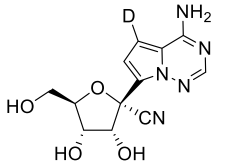	0.39 ± 0.08	> 500
X2	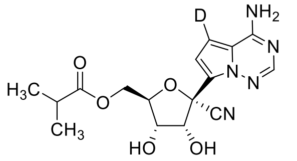	0.25 ± 0.02	> 500
X3	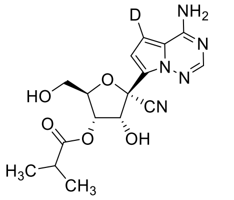	0.27 ± 0.04	> 500
X4	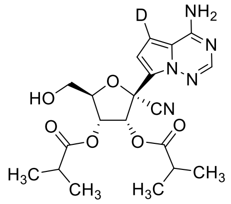	NT	NT
X5	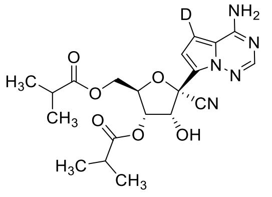	NT	NT
X6	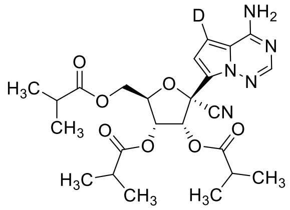	0.35 ± 0.09	280.19 ± 15.39

Supplementary information, Table S3: **Single-dose PK parameters for X1 in rats (for screening).** Calculation of PK parameters for X1 following oral administration of X2, X3 or X6 at 10.0 mg/kg X1 equivalent dose, and intravenous administration at 2.0 mg/kg X1 equivalent dose in SD rats (N = 3 per group).

**Compd. (Route)**	**T_{max} (h)**	**C_{max} (ng/mL)**	**$AUC_{0\text{-}t}$ (h*ng/mL)**	**$AUC_{0\text{-}\infty}$ (h*ng/mL)**	**$MRT_{0\text{-}\infty}$ (h)**	**$T_{1/2}$ (h)**	**CL (mL/min/kg)**	**Vss (L/kg)**	**F (%)**
X1 (p.o.)	2.0 ± 0.0	246 ± 48	1869 ± 412	1912 ± 416	6.44 ± 0.23	4.45 ± 0.43	-	-	21.7
X1 (i.v.)	-	1630 ± 166	1724 ± 130	1789 ± 115	2.41 ± 0.86	6.68 ± 4.76	18.7 ± 1.2	2.67 ± 0.85	
X2 (p.o.)	2.0 ± 0.0	656 ± 94	2556 ± 240	2703 ± 162	4.14 ± 1.12	4.05 ± 2.79	-		32.6
X2 (i.v.)	-	1570 ± 120	1611 ± 204	1657 ± 226	2.62 ± 1.04	5.0 ± 3.52	25.5 ± 3.3	3.87 ± 1.09	
X3 (p.o.)	0.33 ± 0.14	801 ± 97	4455 ± 95	4487 ± 91	4.23 ± 0.22	4.03 ± 0.58	-		46.5
X3 (i.v.)	-	2520 ± 125	1915 ± 47	2009 ± 15	2.43 ± 0.81	6.71 ± 2.48	20.7 ± 0.2	3.02 ± 0.99	
X6 (p.o.)	0.33 ± 0.14	1370 ± 290	4539 ± 511	4717 ± 599	3.10 ± 0.45	1.97 ± 0.27	-	-	49.9
X6 (i.v.)	-	1693 ± 268	1820 ± 87	1832 ± 88	1.35 ± 0.10	1.26 ± 0.07	31.0 ± 1.5	2.50 ± 0.1	

Supplementary information, Table S4: Single-dose PK parameters for X1 in cynomolgus monkey (for screening). Calculation of PK parameters for X1 following oral (10 mg/kg) and intravenous (5 mg/kg) administration of the monoisobutyrate ester prodrug (**X2** and **X3**) in cynomolgus monkeys (N = 3 per group).

**Compd. (Route)**	**$T_{1/2}$ (h)**	**T_{max} (h)**	**C_{max} (ng/mL)**	**AUG_{last} (h*ng/mL)**	**AUG_{INF_obs} (h*ng/mL)**	**$CL_{\_obs}$ (mL/min/kg)**	**MRT_{INF_obs} (h)**	**$Vss_{\_obs}$ (L/kg)**	**F (%)**
X2 (p.o.)	1.95 ± 0.49	1.0 ± 0.0	941 ± 129	1939 ± 388	2069 ± 323	-	2.98 ± 0.55		14.2
X2 (i.v.)	1.31 ± 0.06	-	3322 ± 267	6814 ± 662	6918 ± 682	9.70 ± 0.99	1.74 ± 0.07	1.01 ± 0.1	
X3 (p.o.)	2.25 ± 0.24	1.0 ± 0.0	1064 ± 341	2497 ± 238	2682 ± 247	-	2.97 ± 0.32		18.4
X3 (i.v.)	1.37 ± 0.09	-	7766 ± 968	6780 ± 1104	6857 ± 1108	9.89 ± 1.52	1.32 ± 0.11	0.78 ± 0.11	

Supplementary information, Table S5: The histopathology scores for lung tissues from vehicle-treated and VV116-treated mice[a]

Group	Animal number	Interstitial inflammation	Perivascular/peribronchial infiltration	Perivascular/alveolar edema	Cellular debris	Congestion	Hemorrhage	Percentage of area involved in Interstitial inflammation (%)
Vehicle	V-1	2	1	2	1	1	2	70
	V-4	2	2	2	1	1	1	60
	V-8	1	1	1	1	1	1	35
	V-10	2	2	0	1	1	1	60
VV116 50 mg/kg	116-1	2	2	1	2	1	0	60
	116-2	2	2	1	2	1	1	65
	116-3	3	2	1	2	1	0	80
VV116 100 mg/kg	116-4	1	2	1	1	1	1	30
	116-5	1	1	1	1	1	0	30
	116-6	1	1	1	1	1	0	15

[a] Scored on a scale of 0–3: “0” = none or few, “1” = mild, “2” = moderate, and “3” = severe

Supplementary information, Table S6: Single-dose PK parameters for X1 in SD rats (preclinical PK studies). Calculation of PK parameters for X1 after a single i.v. dose (10 mg/kg) of VV116 in SD rats (N = 6 per group).

**Gender**	**$AUC_{0\text{-}t}$**	**$AUC_{0\text{-}\infty}$**	**$MRT_{0\text{-}\infty}$**	**$t_{1/2}$**	**CL**	**V_{ss}**
	**(ng·h/ml)**	**(ng·h/ml)**	**(h)**	**(h)**	**(ml/min/kg)**	**(L/kg)**
Male	4446 ± 275	4473 ± 267	1.29 ± 0.08	1.11 ± 0.08	37.4 ± 2.3	2.89 ± 0.03
Female	4718 ± 69	4760 ± 68	1.35 ± 0.10	1.77 ± 0.60	35.0 ± 0.5	2.84 ± 0.25
Total	4582 ± 233	4616 ± 235	1.32 ± 0.09	1.44 ± 0.53	36.2 ± 2.0	2.87 ± 0.16

Supplementary information, Table S7: Single-dose PK parameters for X1 in SD rats (preclinical PK studies). Calculation of PK parameters for X1 after single p.o. doses of VV116 in SD rats (N = 6 per group).

Dose mg/kg	Gender	T_{max} (h)	C_{max} (ng/ml)	AUC_{0-t} (ng·h/ml)	$AUC_{0-\infty}$ (ng·h/ml)	$MRT_{0-\infty}$ (h)	$t_{1/2}$ (h)	F %
10	Male	1.0 (0.5-4.0)	608 ± 92	3322 ± 162	3403 ± 142	3.63 ± 0.11	1.51 ± 0.29	
	Female	1.0 (0.5-2.0)	963 ± 136	4598 ± 428	4689 ± 504	3.20 ± 0.60	1.36 ± 0.28	
	Total	1.0 (0.5-4.0)	785 ± 220	3960 ± 757	4046 ± 778	3.42 ± 0.45	1.43 ± 0.27	86.4%
30	Male	1.0 (0.5-4.0)	1563 ± 340	8652 ± 423	9575 ± 312	4.88 ± 0.69	2.85 ± 0.78	
	Female	0.5 (0.5-4.0)	2573 ± 83	13114 ± 244	13342 ± 220	3.46 ± 0.27	1.33 ± 0.15	
	Total	0.75 (0.5-4.0)	2068 ± 596	10883 ± 2463	11458 ± 2077	4.17 ± 0.90	2.09 ± 0.98	79.2%
90	Male	0.5 (0.5-1.0)	5107 ± 1347	33181 ± 3573	33237 ± 3572	5.30 ± 1.04	3.32 ± 1.41	
	Female	2.0 (0.5-4.0)	6973 ± 1221	32433 ± 1162	32533 ± 1221	4.14 ± 0.05	3.96 ± 1.86	
	Total	0.75 (0.5-4.0)	6040 ± 1539	32807 ± 2412	32885 ± 2418	4.72 ± 0.91	3.64 ± 1.52	79.6%

Supplementary information, Table S8: Multidose PK parameters for X1 in SD rats (preclinical PK studies). Calculation of PK parameters for X1 after multiple p.o. doses of VV116 at 30 mg/kg in SD rats (N = 6 per group).

**Dose mg/kg**	**Gender**	**T_{max} (h)**	**C_{max} (ng/ml)**	**AUC_{0-t} (ng·h/ml)**	**$AUC_{0-\infty}$ (ng·h/ml)**	**$MRT_{0-\infty}$ (h)**	**$t_{1/2}$ (h)**
7 × 30	Male	2.0 (1.0-2.0)	1913 ± 210	7917 ± 1082	9519 ± 1372	/	4.15 ± 3.95
	Female	2.0 (1.0-2.0)	1567 ± 145	7569 ± 872	9083 ± 1392	/	3.68 ± 0.94
	Total	2.0 (1.0-2.0)	1740 ± 249	7743 ± 899	9301 ± 1259	/	3.92 ± 2.58

Supplementary information, Table S9: Single-dose PK parameters for X1 in Beagle dogs (preclinical PK studies). Calculation of PK parameters for X1 after a single i.v. dose (10 mg/kg) of VV116 in Beagle dogs (N = 6 per group).

**Gender**	**AUC_{0-t}**	**$AUC_{0-\infty}$**	**$MRT_{0-\infty}$**	**$t_{1/2}$**	**CL**	**V_{ss}**
	**(ng·h/ml)**	**(ng·h/ml)**	**(h)**	**(h)**	**(ml/min/kg)**	**(L/kg)**
Male	14697 ± 3569	14934 ± 3451	3.60 ± 0.75	3.54 ± 1.09	11.6 ± 2.8	2.42 ± 0.06
Female	16973 ± 2150	17168 ± 2187	4.10 ± 0.33	4.34 ± 0.37	9.82 ± 1.34	2.41 ± 0.20
Total	15835 ± 2916	16051 ± 2859	3.85 ± 0.58	3.94 ± 0.85	10.7 ± 2.2	2.41 ± 0.13

Supplementary information, Table S10: Single-dose PK parameters for X1 in Beagle dogs (preclinical PK studies). Calculation of PK parameters for X1 after single p.o. doses of VV116 in Beagle dogs (N = 6 per group).

**Dose**	**Gender**	$\mathbf{T_{max}}$	$\mathbf{C_{max}}$	$\mathbf{AUC_{0\text{-}t}}$	$\mathbf{AUC_{0\text{-}\infty}}$	$\mathbf{MRT_{0\text{-}\infty}}$	$\mathbf{t_{1/2}}$	**F**
**mg/kg**		**(h)**	**(ng/ml)**	**(ng·h/ml)**	**(ng·h/ml)**	**(h)**	**(h)**	**%**
10	Male	1.17 ± 0.76	3407 ± 404	13801 ± 3550	14015 ± 3503	4.38 ± 0.61	4.98 ± 1.33	
	Female	0.83 ± 0.29	3030 ± 960	13889 ± 2895	14312 ± 2939	4.83 ± 1.50	4.07 ± 1.86	
	Total	1.00 ± 0.55	3218 ± 690	13845 ± 2898	14163 ± 2896	4.61 ± 1.05	4.53 ± 1.53	87.4%
20	Male	0.83 ± 0.29	7143 ± 301	30010 ± 3992	30365 ± 4085	4.52 ± 0.16	4.16 ± 0.25	
	Female	1.33 ± 0.58	6123 ± 2208	34401 ± 4003	34968 ± 4094	5.40 ± 0.61	4.25 ± 1.13	
	Total	1.08 ± 0.49	6633 ± 1516	32206 ± 4309	32667 ± 4442	4.96 ± 0.63	4.21 ± 0.73	101.7%
40	Male	0.83 ± 0.29	11693 ± 5519	56111 ± 13640	56754 ± 13685	4.94 ± 0.84	4.02 ± 0.46	
	Female	1.33 ± 0.58	10423 ± 3978	70468 ± 5504	71115 ± 5753	6.23 ± 1.93	4.52 ± 0.88	
	Total	1.08 ± 0.49	11058 ± 4359	63289 ± 12181	63935 ± 12248	5.59 ± 1.51	4.27 ± 0.68	99.9%

Supplementary information, Table S11: Multidose PK parameters for 116-N1 in Beagle dogs (preclinical PK studies). Calculation of PK parameters for X1 after multiple p.o. doses of VV116 at 20 mg/kg in Beagle dogs (N = 6 per group).

Dose mg/kg	Gender	T_{max} (h)	C_{max} (ng/ml)	AUC_{0-t} (ng·h/ml)	$AUC_{0-\infty}$ (ng·h/ml)	$MRT_{0-\infty}$ (h)	$t_{1/2}$ (h)
7 × 20	Male	1.33 ± 0.58	5727 ± 2138	26956 ± 4076	27258 ± 4196	/	4.03 ± 0.24
	Female	1.00 ± 0.87	6907 ± 3478	27788 ± 3360	28268 ± 3411	/	4.65 ± 0.27
	Total	1.17 ± 0.68	6317 ± 2662	27372 ± 3372	27763 ± 3464	/	4.34 ± 0.41

Supplementary information, Table S12: Metabolites identified in SD rats and Beagle dogs

Metabolites	metabolic pathway	Molecular formula	[M-H]⁻(m/z)	Structure	SD rats	Beagle dogs
X1	hydrolysis	$C_{12}DH_{12}N_5O_4$	291.0958	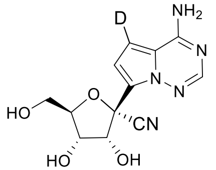	primary metabolite	primary metabolite
M1	ring opening and oxidation	$C_{11}DH_{13}N_4O_5$	282.0942	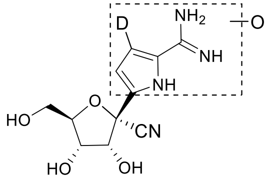	ND	detected in plasma
M2	deamination and oxidation	$C_{12}DH_{11}N_4O_5$	292.0789	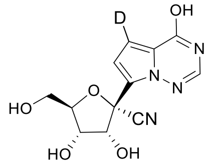	detected in plasma, urine and feces	detected in plasma
M3	ring opening and glycine conjugation	$C_{13}DH_{16}N_5O_5$	323.1205	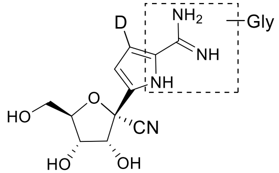	detected in plasma	detected in plasma
M4-1	sulfonation	$C_{12}DH_{12}N_5O_7S$	371.0515	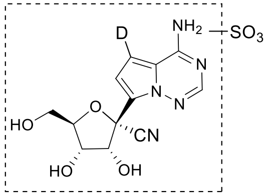	detected in urine, feces and bile	ND

M4-2	sulfonation	$C_{12}DH_{12}N_5O_7S$	371.0513	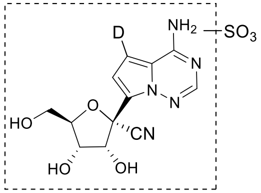	detected in plasma, urine, feces and bile	ND
M5	sulfonation and oxidation	$C_{12}DH_{12}N_5O_8S$	387.045	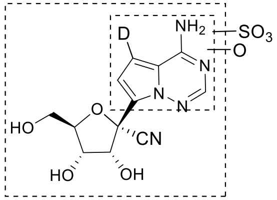	ND	detected in plasma
M6-1	glucuronide conjugation	$C_{18}DH_{20}N_5O_{10}$	467.125	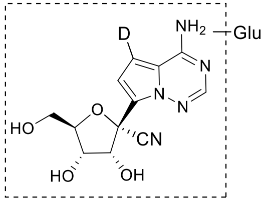	detected in plasma and bile	detected in plasma
M6-2	glucuronide conjugation	$C_{18}DH_{20}N_5O_{10}$	467.1268	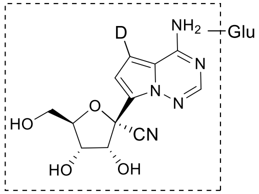	detected in plasma, urine and bile	ND

"ND" = "Not Detected"
